# Polygenic Modeling with Bayesian Sparse Linear Mixed Models

**DOI:** 10.1371/journal.pgen.1003264

**Published:** 2013-02-07

**Authors:** Xiang Zhou, Peter Carbonetto, Matthew Stephens

**Affiliations:** 1 Department of Human Genetics, University of Chicago, Chicago, Illinois, United States of America; 2 Department of Statistics, University of Chicago, Chicago, Illinois, United States of America; The University of Queensland, Australia

## Abstract

Both linear mixed models (LMMs) and sparse regression models are widely used in genetics applications, including, recently, polygenic modeling in genome-wide association studies. These two approaches make very different assumptions, so are expected to perform well in different situations. However, in practice, for a given dataset one typically does not know which assumptions will be more accurate. Motivated by this, we consider a hybrid of the two, which we refer to as a “Bayesian sparse linear mixed model” (BSLMM) that includes both these models as special cases. We address several key computational and statistical issues that arise when applying BSLMM, including appropriate prior specification for the hyper-parameters and a novel Markov chain Monte Carlo algorithm for posterior inference. We apply BSLMM and compare it with other methods for two polygenic modeling applications: estimating the proportion of variance in phenotypes explained (PVE) by available genotypes, and phenotype (or breeding value) prediction. For PVE estimation, we demonstrate that BSLMM combines the advantages of both standard LMMs and sparse regression modeling. For phenotype prediction it considerably outperforms either of the other two methods, as well as several other large-scale regression methods previously suggested for this problem. Software implementing our method is freely available from http://stephenslab.uchicago.edu/software.html.

## Author Summary

The goal of polygenic modeling is to better understand the relationship between genetic variation and variation in observed characteristics, including variation in quantitative traits (e.g. cholesterol level in humans, milk production in cattle) and disease susceptibility. Improvements in polygenic modeling will help improve our understanding of this relationship and could ultimately lead to, for example, changes in clinical practice in humans or better breeding/mating strategies in agricultural programs. Polygenic models present important challenges, both at the modeling/statistical level (what modeling assumptions produce the best results) and at the computational level (how should these models be effectively fit to data). We develop novel approaches to help tackle both these challenges, and we demonstrate the gains in accuracy that result in both simulated and real data examples.

## Introduction

Both linear mixed models (LMMs) and sparse regression models are widely used in genetics applications. For example, LMMs are often used to control for population stratification, individual relatedness, or unmeasured confounding factors when performing association tests in genetic association studies [1]–[9] and gene expression studies [10]–[12]. They have also been used in genetic association studies to jointly analyze groups of SNPs [13], [14]. Similarly, sparse regression models have been used in genome-wide association analyses [15]–[20] and in expression QTL analysis [21]. Further, both LMMs and sparse regression models have been applied to, and garnered renewed interest in, polygenic modeling in association studies. Here, by polygenic modeling we mean any attempt to relate phenotypic variation to many genetic variants simultaneously (in contrast to single-SNP tests of association). The particular polygenic modeling problems that we focus on here are estimating “chip heritability”, being the proportion of variance in phenotypes explained (PVE) by available genotypes [19], [22]–[24], and predicting phenotypes based on genotypes [25]–[29].

Despite the considerable overlap in their applications, in the context of polygenic modeling, LMMs and sparse regression models are based on almost diametrically opposed assumptions. Precisely, applications of LMMs to polygenic modeling (e.g. [22]) effectively assume that every genetic variant affects the phenotype, with effect sizes normally distributed, whereas sparse regression models, such as Bayesian variable selection regression models (BVSR) [18], [19], assume that a relatively small proportion of all variants affect the phenotype. The relative performance of these two models for polygenic modeling applications would therefore be expected to vary depending on the true underlying genetic architecture of the phenotype. However, in practice, one does not know the true genetic architecture, so it is unclear which of the two models to prefer. Motivated by this observation, we consider a hybrid of these two models, which we refer to as the “Bayesian sparse linear mixed model”, or BSLMM. This hybrid includes both the LMM and a sparse regression model, BVSR, as special cases, and is to some extent capable of learning the genetic architecture from the data, yielding good performance across a wide range of scenarios. By being “adaptive” to the data in this way, our approach obviates the need to choose one model over the other, and attempts to combine the benefits of both.

The idea of a hybrid between LMM and sparse regression models is, in itself, not new. Indeed, models like these have been used in breeding value prediction to assist genomic selection in animal and plant breeding programs [30]–[35], gene selection in expression analysis while controlling for batch effects [36], phenotype prediction of complex traits in model organisms and dairy cattle [37]–[40], and more recently, mapping complex traits by jointly modeling all SNPs in structured populations [41]. Compared with these previous papers, our work makes two key contributions. First, we consider in detail the specification of appropriate prior distributions for the hyper-parameters of the model. We particularly emphasize the benefits of estimating the hyper-parameters from the data, rather than fixing them to pre-specified values to achieve the adaptive behavior mentioned above. Second, we provide a novel computational algorithm that exploits a recently described linear algebra trick for LMMs [8], [9]. The resulting algorithm avoids *ad hoc* approximations that are sometimes made when fitting these types of model (e.g. [37], [41]), and yields reliable results for datasets containing thousands of individuals and hundreds of thousands of markers. (Most previous applications of this kind of model involved much smaller datasets.)

Since BSLMM is a hybrid of two widely used models, it naturally has a wide range of potential uses. Here we focus on its application to polygenic modeling for genome-wide association studies, specifically two applications of particular interest and importance: PVE estimation (or “chip heritability” estimation) and phenotype prediction. Estimating the PVE from large-scale genotyped marker sets (e.g. all the SNPs on a genome-wide genotyping chip) has the potential to shed light on sources of “missing heritability” [42] and the underlying genetic architecture of common diseases [19], [22]–[24], [43]. And accurate prediction of complex phenotypes from genotypes could ultimately impact many areas of genetics, including applications in animal breeding, medicine and forensics [27]–[29], [37]–[40]. Through simulations and applications to real data, we show that BSLMM successfully combines the advantages of both LMMs and sparse regression, is robust to a variety of settings in PVE estimation, and outperforms both models, and several related models, in phenotype prediction.

## Methods

### Background and Motivation

We begin by considering a simple linear model relating phenotypes 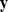 to genotypes 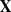:
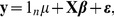
(1)

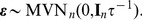
(2)


Here 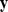 is an 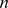-vector of phenotypes measured on 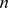 individuals, 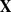 is an 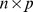 matrix of genotypes measured on the same individuals at 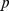 genetic markers, 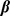 is a 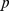-vector of (unknown) genetic marker effects, 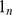 is an 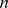-vector of 1 s, 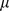 is a scalar representing the phenotype mean, and 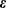 is an 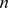-vector of error terms that have variance 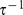. 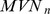 denotes the 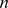-dimensional multivariate normal distribution. Note that there are many ways in which measured genotypes can be encoded in the matrix 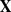. We assume throughout this paper that the genotypes are coded as 0, 1 or 2 copies of a reference allele at each marker, and that the columns of 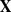 are centered but not standardized; see Text S1.

A key issue is that, in typical current datasets (e.g. GWAS), the number of markers 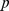 is much larger than the number of individuals 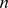. As a result, parameters of interest (e.g. 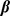 or PVE) cannot be estimated accurately without making some kind of modeling assumptions. Indeed, many existing approaches to polygenic modeling can be derived from (1) by making specific assumptions about the genetic effects 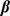. For example, the LMM approach from [22], which has recently become commonly used for PVE estimation (e.g. [24], [44]–[46]), is equivalent to the assumption that effect sizes are normally distributed, such that
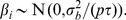
(3)[Specifically, exact equivalence holds when the relatedness matrix in the LMM is computed from the genotypes as 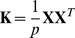 (e.g. [47]). [22] use a matrix in this form, with 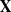 centered and standardized, and with a slight modification of the diagonal elements.] For brevity, in this paper we refer to the regression model that results from this assumption as the LMM (note that this is relatively restrictive compared with the usual definition); it is also commonly referred to as “ridge regression” in statistics [48]. The estimated combined effects (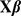), or equivalently, the estimated random effects, obtained from this model are commonly referred to as Best Linear Unbiased Predictors (BLUP) [49].

An alternative assumption, which has also been widely used in polygenic modeling applications [18], [19], [34], and more generally in statistics for sparse high-dimensional regression with large numbers of covariates [50], [51], is that the effects come from a mixture of a normal distribution and a point mass at 0, also known as a point-normal distribution:
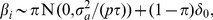
(4)where 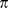 is the proportion of non-zero 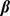 and 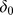 denotes a point mass at zero. [This definition of 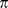 follows the convention from statistics [19], [50], [51], which is opposite to the convention in animal breeding [27], [32]–[34], [40].] We refer to the resulting regression model as Bayesian Variable Selection Regression (BVSR), because it is commonly used to select the relevant variables (i.e. those with non-zero effect) for phenotype prediction. Although (4) formally includes (3) as a special case when 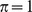, in practice (4) is often used together with an assumption that only a small proportion of the variables are likely to be relevant for phenotype prediction, say by specifying a prior distribution for 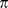 that puts appreciable mass on small values (e.g. [19]). In this case, BVSR and LMM can be viewed as making almost diametrically opposed assumptions: the LMM assumes every variant has an effect, whereas BVSR assumes that a very small proportion of variants have an effect. (In practice, the estimate of 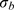 under LMM is often smaller than the estimate of 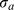 under BVSR, so we can interpret the LMM as assuming a large number of small effects, and BVSR as assuming a small number of larger effects.)

A more general assumption, which includes both the above as special cases, is that the effects come from a mixture of two normal distributions:
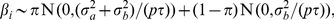
(5)Setting 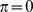 yields the LMM (3), and 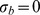 yields BVSR (4). we can interpret this model as assuming that all variants have at least a small effect, which are normally distributed with variance 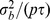, and some proportion (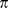) of variants have an additional effect, normally distributed with variance 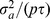. The earliest use of a mixture of two normal distributions for the regression coefficients that we are aware of is [52], although in that paper various hyper-parameters were fixed, and so it did not include LMM and BVSR as special cases.

Of the three assumptions on the effect size distributions above, the last (5) is clearly the most flexible since it includes the others as special cases. Obviously other assumptions are possible, some still more flexible than the mixture of two normals: for example, a mixture of three or more normals. Indeed, many other assumptions have been proposed, including variants in which a normal distribution is replaced by a 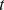 distribution. These variants include the “Bayesian alphabet models” – so-called simply because they have been given names such as BayesA, BayesB, BayesC, etc. – that have been proposed for polygenic modeling, particularly breeding value prediction in genomic selection studies. Table 1 summarizes these models, and some other effect size distributions that have been proposed, together with relevant references (see also [53] and the references there in). Among these, the models most closely related to ours are BayesC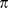 [34] and BayesR [35]. Specifically, BayesC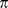 without a random effect is BVSR, and with a random effect is BSLMM (which we define below). BayesR models effect sizes using a mixture of three normal components plus a point mass at zero, although the relative variance for each normal distribution is fixed.

**Table 1:** Summary of some effect size distributions that have been proposed for polygenic modeling.

Effect Size Distribution		Keywords	Selected References
Name	Formula		
t	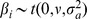	BayesA	[27], [32], [33], [40]
point-t	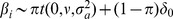	BayesB, BayesD, BayesD 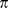	[27], [32]–[34], [40]
t mixture	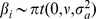 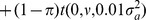	BayesC	[32], [33]
point-normal	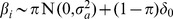	BayesC, BayesC 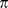 , BVSR	[18], [19], [34]
double exponential	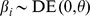	Bayesian Lasso	[28], [39], [68]
point-normal mixture	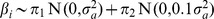 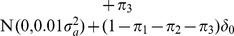	BayesR	[35]
normal	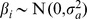	LMM, BLUP, Ridge Regression	[22], [26], [28], [48]
normal-exponential-gamma	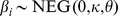	NEG	[16]
normal mixture	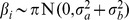 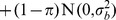	BSLMM	Present Work

The reference list contains only a selection of relevant publications. Abbreviations: DE denotes double exponential distribution, NEG denotes normal exponential gamma distribution, and other abbreviations can be found in the main text. In the scaled t-distribution, 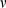 and 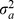 are the degree of freedom parameter and scale parameter, respectively. In the DE distribution, 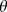 is the scale parameter. In the NEG distribution, 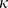 and 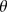 are the shape and scale parameters, respectively. Notes: 1. Some applications of these methods combine a particular effect size distribution with a random effects term, with covariance matrix 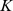, to capture sample structure or relatedness. If 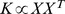 then this is equivalent to adding a normal distribution to the effect size distribution. The listed effect size distributions in this table do not include this additional normal component. 2. BayesC has been used to refer to models with different effect size distributions in different papers. 3. In some papers, keywords listed here have been used to refer to fitting techniques rather than effect size distributions.

Given the wide range of assumptions for effect size distributions that have been proposed, it is natural to wonder which are the most appropriate for general use. However, answering this question is complicated by the fact that even given the effect size distribution there are a number of different ways that these models can be implemented in practice, both in terms of statistical issues, such as treatment of the hyper-parameters, and in terms of computational and algorithmic issues. Both these types of issues can greatly affect practical performance. For example, many approaches fix the hyper-parameters to specific values [27], [32], [33], [40] which makes them less flexible [34], [54]. Thus, in this paper we focus on a particular effect size distribution (5), which while not the most flexible among all that could be considered, is certainly more flexible than the one that has been most used in practice for estimating PVE (LMM), and admits certain computational methods that could not be applied in all cases. We consider in detail how to apply this model in practice, and the resulting advantages over LMM and BVSR (although we also compare with some other existing approaches). A key contribution of this paper is to provide new approaches to address two important practical issues: the statistical issue of how to deal with the unknown hyper-parameters 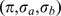, and the computational issue of how to fit the model. Notably, with the computational tools we use here, fitting the model (5) becomes, for a typical dataset, less computationally intensive than fitting BVSR, as well as providing gains in performance.

With this background, we now turn to detailed description of the model, its prior specification and its computation algorithm.

### The Bayesian Sparse Linear Mixed Model

In this paper we focus on the simple linear model (1) with mixture prior (5) on the effects. However, the computational and statistical methods we use here also apply to a more general model, which we refer to as the Bayesian Sparse Linear Mixed Model (BSLMM), and which includes the model (1) with (5) as a special case.

The BSLMM consists of a standard linear mixed model, with one random effect term, and with sparsity inducing priors on the regression coefficients:
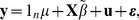
(6)

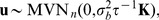
(7)

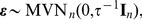
(8)

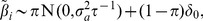
(9)where 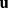 is an 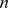-vector of random effects with known 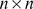 covariance matrix 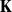. In referring to 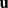 as the “random effects” we are following standard terminology from LMMs. Standard terminology also refers to the coefficients 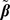 as “fixed effects”, but this phrase has a range of potential meanings [55] and so we avoid it here. Instead we use the term “sparse effects” for these parameters to emphasize the sparsity-inducing prior.

It is straightforward to show that when 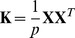, BSLMM is equivalent to the simple linear model (1) with mixture prior (5) on the effects. However, our discussion of prior specification, computational algorithms, and software, all apply for any 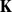.

When we say that (6) is equivalent to (1) with (5), this equivalence refers to the implied probability model for 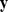 given 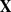 and the hyper-parameters 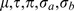. However, 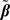 and 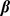 are not equivalent (explaining our use of two different symbols): in (6) the random effect 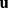 captures the combined small effects of all markers, whereas in (1) these small effects are included in 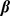. Since both our applications focus on the relationship between 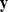 and 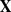, and not on interpreting estimates of 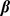 or 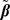, we do not concern ourselves any further with this issue, although it may need consideration in applications where individual estimated genetic effects are of more direct interest (e.g. genetic association mapping). A related issue is the interpretation of the random effect 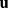 in BSLMM: from the way we have presented the material 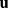 is most naturally interpreted as representing a polygenic component, specifically the combined effect of a large number of small effects across all measured markers. However, if there are environmental factors that influence the phenotype and are correlated with genotype (e.g. due to population structure), then these would certainly affect estimates of 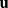, and consequently also affect estimates of other quantities, including the PVE. In addition, phenotype predictions from BSLMM will include a component due to unmeasured environmental factors that are correlated with measured genotypes. These issues are, of course, not unique to BSLMM – indeed, they apply equally to the LMM; see [56] and the response from [57] for relevant discussion.

Finally, given the observation that a mixture of two normals is more flexible than a point-normal, it might seem natural to consider modifying (6) by making the assumption that 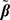 comes from a mixture of two normal distributions rather than a point-normal. However, if 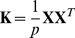 then this modification is simply equivalent to changing the values of 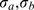.

### Prior Specification

The BSLMM involves (hyper-)parameters, 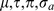, and 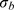. Before considering prior specification for these parameters, we summarize their interpretations as follows:

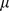
 and 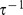 control the phenotype mean and residual variance.

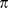
 controls the proportion of 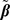 values in (6) that are non-zero.

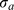
 controls the expected magnitude of the (non-zero) 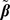.

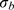
 controls the expected magnitude of the random effects 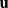.

Appropriate values for these parameters will clearly vary for different datasets, so it seems desirable to estimate them from the data. Here we accomplish this in a Bayesian framework by specifying prior distributions for the parameters, and using Markov chain Monte Carlo (MCMC) to obtain approximate samples from their posterior distribution given the observed data. Although one could imagine instead using maximum likelihood to estimate the parameters, the Bayesian framework has several advantages here: for example, it allows for incorporation of external information (e.g. that most genetic markers will, individually, have small effects), and it takes into account of uncertainty in parameter estimates when making other inferences (e.g. phenotype prediction).

For the mean 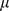 and the inverse of error variance, 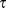, we use the standard conjugate prior distributions:
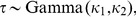
(10)

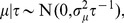
(11)where 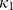 and 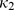 denote, respectively, shape and rate parameters of a Gamma distribution. Specifically we consider the posterior that arises in the limits 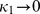, 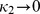 and 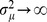. These limits correspond to improper priors, but the resulting posteriors are proper, and scale appropriately with shifting or scaling of the phenotype vector 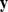 [58]. In particular, these priors have the property that conclusions will be unaffected by changing the units of measurement of the phenotype, which seems desirable for a method intended for general application.

Prior specification for the remaining hyper-parameters 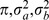 is perhaps more important. Our approach is to extend the prior distributions for BVSR described in [19].

Following [19] we place a uniform prior on 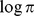:
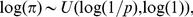
(12)where 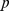 is total number of markers being analyzed. The upper and lower limit of this prior were chosen so that 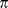 (the expected proportion of markers with non-zero 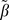) ranges from 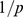 to 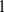. A uniform prior on 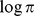 reflects the fact that uncertainty in 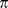 in a typical GWAS will span orders of magnitude. A common alternative (see e.g. [18], [34]) is a uniform distribution on 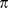, but as noted in [19] this puts large weight on large numbers of markers having non-zero 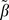 (e.g. it would correspond to placing 50% prior probability to the event that more than half of the markers have non-zero 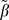, and correspond to placing 90% prior probability to the event that more than 10% of the markers have non-zero 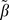).

To specify priors for 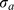 and 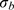, we exploit the following idea from [19]: prior specification is easier if we first re-parameterize the model in terms of more interpretable quantities. Specifically we extend ideas from [19] to re-parameterize the model in terms of the (expected) proportion of phenotypic variance explained by the sparse effects and by the random effects.

To this end, we define PVE (the total proportion of variance in phenotype explained by the sparse effects and random effects terms together) and PGE (the proportion of genetic variance explained by the sparse effects terms) as functions of 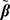, 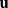 and 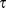:
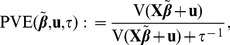
(13)

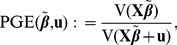
(14)where the function V(x) is defined as
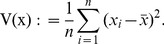
(15)These definitions ensure that both PVE and PGE must lie in the interval 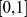. PVE reflects how well one could predict phenotype 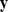 from the available SNPs if one knew the optimal 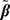 as well as the random effects 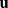; together with PVE, PGE reflects how well one could predict phenotype 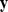 using 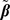 alone.

Since PVE and PGE are functions of 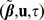, whose distributions depend on hyper-parameters 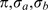, the prior distribution for PVE and PGE depends on the priors assigned to these hyper-parameters. In brief, our aim is to choose priors for the two hyper-parameters 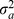 and 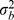 so that the induced priors on both PVE and PGE are roughly uniform on 0 and 1. (Other distributions could be chosen if desired, but we consider this uniform distribution one reasonable default.) However, because the relationship between the distribution of PVE, PGE and the hyper-parameters is not simple, we have to make some approximations.

Specifically, we introduce 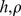 as approximations (they are ratios of expectations rather than expectations of ratios) to the expectations of PVE and PGE, respectively:
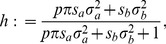
(16)

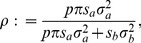
(17)where 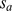 is the average variance of genotypes across markers, and 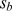 is the mean of diagonal elements in 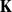. In other words, 
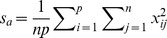
 and 
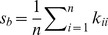
, where 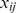 and 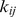 are the 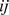
th elements of matrices 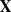 and 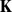, respectively. See Text S1 for derivations. Intuitively, the term 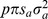 captures the expected genetic variance contributed by the sparse effects term (relative to the error variance), because 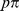 is the expected number of causal markers, 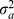 is the expected effect size variance of each causal marker (relative to the error variance), and 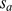 is the average variance of marker genotypes. Similarly, the term 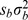 captures the expected genetic variance contributed by the random effects term (relative to the error variance), because 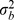 is the expected variance of the random effects (relative to the error variance) when the relatedness matrix has unit diagonal elements, while 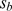 properly scales it when not.

The parameter 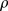 provides a natural bridge between the LMM and BVSR: when 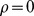 BSLMM becomes the LMM, and when 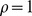 BSLMM becomes BVSR. In practice, when the data favors the LMM, the posterior distribution of 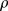 would mass near 0, and when the data favors BVSR, the posterior distribution of 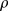 would mass near 1.

In summary, the above re-parameterizes the model in terms of 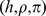 instead of 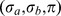. Now, instead of specifying prior distributions for 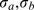, we rather specify prior distributions for 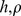. Specifically we use uniform prior distributions for 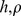:
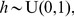
(18)

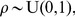
(19)independent of one another and of 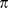. Since 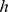 and 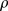 approximate PVE and PGE, these prior distributions should lead to reasonably uniform prior distributions for PVE and PGE, which we view as reasonable defaults for general use. (If one had specific information about PVE and PGE in a given application then this could be incorporated here.) In contrast it seems much harder to directly specify reasonable default priors for 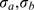 (although these priors on 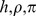 do of course imply priors for 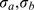; see Text S1).

Note that we treat 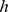 and 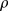 as approximations to PVE and PGE only for prior specification; when estimating PVE and PGE from data we do so directly using their definitions (13) and (14) (see below for details).

### Posterior Sampling Scheme

To facilitate computation, we use the widely-used approach from [52] of introducing a vector of binary indicators 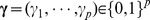 that indicates whether the corresponding coefficients 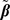 are non-zero. The point-normal priors for 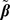 can then be written
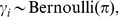
(20)

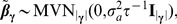
(21)

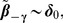
(22)where 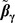 denotes the sub-vector of 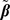 corresponding to the entries 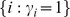; 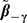 denotes the sub-vector of 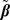 corresponding to the other entries, 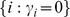; and 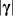 denotes the number of non-zero entries in 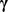. We use MCMC to obtain posterior samples of (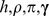) on the product space 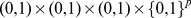, which is given by
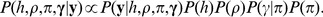
(23)The marginal likelihood 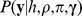 can be computed analytically integrating out 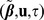; see below for further details. We use a Metropolis-Hastings algorithm to draw posterior samples from the above marginal distribution. In particular, we use a rank based proposal distribution for 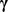 [19], which focus more of the computational time on examining SNPs with stronger marginal associations.

We use the resulting sample from the posterior distribution (23) to estimate PVE and PGE as follows. For each sampled value of 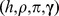, we sample a corresponding value for 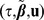 from the conditional distribution 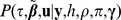. We then use each sampled value of 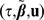 to compute a sampled value of PVE and PGE, using equations (13) and (14). We estimate the posterior mean and standard deviation of PVE, PGE, from these samples.

The novel part of our algorithm is a new efficient approach to evaluating the likelihood 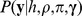 that considerably reduces the overall computational burden of the algorithm. The direct naive approach to evaluating this likelihood involves a matrix inversion and a matrix determinant calculation that scale cubically with the number of individuals 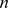, and this cost is incurred every iteration as hyper parameter values change. Consequently, this approach is impractical for typical association studies with large 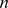, and *ad hoc* approximations are commonly used to reduce the burden. For example, both [37] and [41] fix 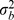 to some pre-estimated value. As we show later, this kind of approximation can reduce the accuracy of predicted phenotypes. Here, we avoid such approximations by exploiting recently developed computational tricks for LMMs [8], [9]. The key idea is to perform a single eigen-decomposition and use the resulting unitary matrix (consisting of all eigen vectors) to transform both phenotypes and genotypes to make the transformed values follow independent normal distributions. By extending these tricks to BSLMM we evaluate the necessary likelihoods much more efficiently. Specifically, after a single 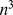 operation at the start of the algorithm, the per iteration computational burden is linear in 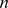 (the same as BVSR), allowing large studies to be analyzed.

Full details of the sampling algorithm appear in Text S2.

### URLs

Software implementing our methods is included in the GEMMA software package, which is freely available at http://stephenslab.uchicago.edu/software.html.

## Results

### Simulations: PVE Estimation

Both the LMM and BVSR have been used to estimate the PVE [19], [22]. Since the LMM assumes that all SNPs have an effect, while BVSR assumes that only a small proportion of SNPs have an effect, we hypothesize that BVSR will perform better when the true underlying genetic structure is sparse and LMM will perform better when the true genetic structure is highly polygenic. Further, because BSLMM includes both as special cases, we hypothesize that BSLMM will perform well in either scenario.

To test these hypotheses, we perform a simulation using real genotypes at about 300,000 SNPs in 3,925 Australian individuals [22], and simulate phenotypes under two different scenarios. In Scenario I we simulate a fixed number 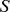 of causal SNPs (with 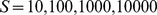), with effect sizes coming from a standard normal distribution. These simulations span a range of genetic architectures, from very sparse to highly polygenic. In Scenario II we simulate two groups of causal SNPs, the first group containing a small number of SNPs of moderate effect (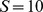 or 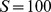), plus a second larger group of 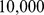 SNPs of small effect representing a “polygenic component”. This scenario might be considered more realistic, containing a mix of small and larger effects. For both scenarios we added normally-distributed errors to phenotype to create datasets with true PVE = 0.6 and 0.2 (equation 13). We simulate 20 replicates in each case, and run the algorithms with all SNPs, including the simulated causal variants, so that the true PVE for typed markers is either 0.6 or 0.2 (if we excluded the causal variants then the true PVE would be unknown).

**Figure 1:**
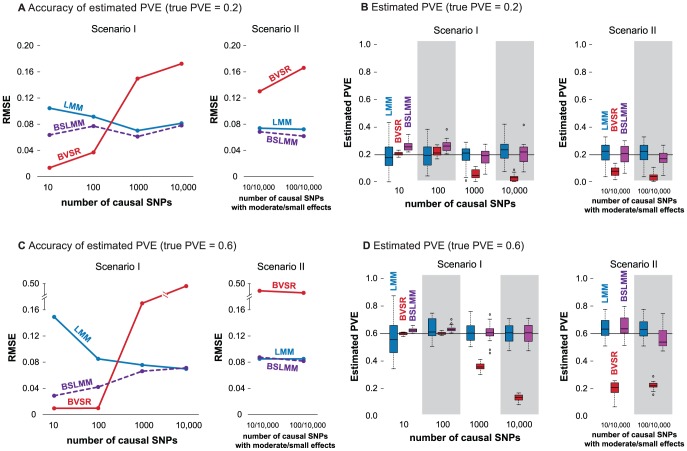
Comparison of PVE estimates from LMM (blue), BVSR (red), and BSLMM (purple) in two simulation scenarios. The x-axis show the number of causal SNPs (Scenario I) or the number of medium/small effect SNPs (Scenario II). Results are based on 20 replicates in each case. (A) (true PVE = 0.2) and (C) (true PVE = 0.6) show RMSE of PVE estimates. (B) (true PVE = 0.2) and (D) (true PVE = 0.6) show boxplots of PVE estimates, where the true PVE is shown as a horizontal line. Notice a break point on the y-axis in (C).

Figure 1A and 1C, show the root of mean square error (RMSE) of the PVE estimates obtained by each method, and Figure 1B and 1D summarize the corresponding distributions of PVE estimates. In agreement with our original hypotheses, BVSR performs best (lowest RMSE) when the true model is sparse (e.g. Scenario I, 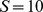 or 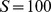 in Figure 1A, 1C). However, it performs very poorly under all the other, more polygenic, models. This is due to a strong downward bias in its PVE estimates (Figure 1B, 1D). Conversely, under the same scenarios, LMM is the least accurate method. This is because the LMM estimates have much larger variance than the other methods under these scenarios (Figure 1B,1D), although, interestingly, LMM is approximately unbiased even in these settings where its modeling assumptions are badly wrong. As hypothesized, BSLMM is robust across a wider range of settings than the other methods: its performance lies between LMM and BVSR when the true model is sparse, and provides similar accuracy to LMM when not.

Of course, in practice, one does not know in advance the correct genetic architecture. This makes the stable performance of BSLMM across a range of settings very appealing. Due to the poor performance of BVSR under highly polygenic models, we would not now recommend it for estimating PVE in general, despite its good performance when its assumptions are met.

### Simulations: Phenotype Prediction

We also compare the three methods on their ability to predict phenotypes from genotypes, using the same simulations.

To measure prediction performance, we use relative prediction gain (RPG; see Text S1). In brief, RPG is a standardized version of mean square error: RPG = 1 when accuracy is as good as possible given the simulation setup, and RPG = 0 when accuracy is the same as simply predicting everyone to have the mean phenotype value. RPG can be negative if accuracy is even worse than that.

**Figure 2:**
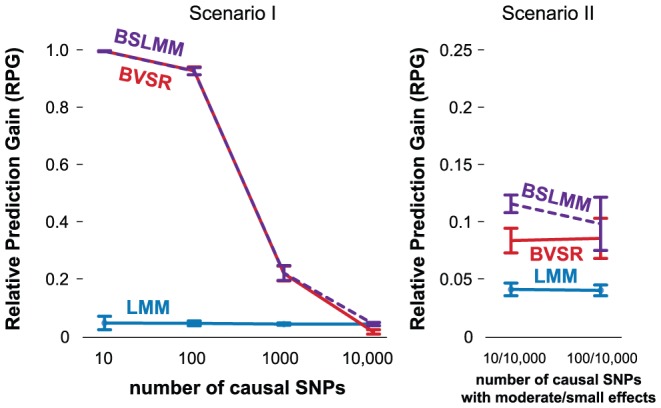
Comparison of prediction performance of LMM (blue), BVSR (red), and BSLMM (purple) in two simulation scenarios, where all causal SNPs are included in the data. Performance is measured by Relative Predictive Gain (RPG). True PVE = 0.6. Means and standard deviations (error bars) are based on 20 replicates. The x-axis show the number of causal SNPs (Scenario I) or the number of medium/small effect SNPs (Scenario II).

Figure 2 compares RPG of different methods for simulations with PVE = 0.6 (results for PVE = 0.2 are qualitatively similar, not shown). Interestingly, for phenotype prediction, the relative performance of the methods differs from results for PVE estimation. In particular, LMM performs poorly compared with the other two methods in all situations, except for Scenario I with 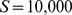, the Scenario that comes closest to matching the underlying assumptions of LMM. As we expect, BSLMM performs similarly to BVSR in scenarios involving smaller numbers of causal SNPs (up to 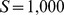 in Scenario I), and outperforms it in more polygenic scenarios involving large numbers of SNPs of small effect (e.g. Scenario II). This is presumably due to the random effect in BSLMM that captures the polygenic component, or, equivalently, due to the mixture of two normal distributions in BSLMM that better captures the actual distribution of effect sizes. The same qualitative patterns hold when we redo these simulation comparisons excluding the causal SNPs (Figure S1) or use correlation instead of RPG to assess performance (Figure S2 and Figure S3).

For a potential explanation why LMM performs much less well for phenotype prediction than for PVE estimation, we note the difference between these two problems: to accurately estimate PVE it suffices to estimate the “average” effect size reliably, whereas accurate phenotype prediction requires accurate estimates of individual effect sizes. In situations where the normal assumption on effect sizes is poor, LMM tends to considerably underestimate the number of large effects, and overestimate the number of small effects. These factors can cancel one another out in PVE estimation, but both tend to reduce accuracy of phenotype prediction.

### Estimating PVE in Complex Human Traits

To obtain further insights into differences between LMM, BVSR and BSLMMM, we apply all three methods to estimate the PVE for five traits in two human GWAS datasets. The first dataset contains height measurements of 3,925 Australian individuals with about 300,000 typed SNPs. The second dataset contains four blood lipid measurements, including high-density lipoprotein (HDL), low-density lipoprotein (LDL), total cholesterol (TC) and triglycerides (TG) from 1,868 Caucasian individuals with about 550,000 SNPs. The narrow sense heritability for height is estimated to be 0.8 from twin-studies [22], [59]. The narrow sense heritabilities for the lipid traits have been estimated, in isolated founder populations, to be 0.63 for HDL, 0.50 for LDL, 0.37 for TG in Hutterites [60], and 0.49 for HDL, 0.42 for LDL, 0.42 for TC and 0.32 for TG in Sardinians [61].

Table 2 shows PVE estimates from the three methods for the five traits. PVE estimates from BVSR are consistently much smaller than those obtained by LMM and BSLMM, which are almost identical for two traits and similar for the others. Estimates of PVE from both LMM and BSLMM explain over 50% of the narrow sense heritability of the five traits, suggesting that a sizable proportion of heritability of these traits can be explained, either directly or indirectly, by available SNPs.

**Table 2:** PVE and PGE estimates for five human traits.

	Method	Height	HDL	LDL	TC	TG
PVE	LMM	0.42 (0.08)	0.38 (0.15)	0.22 (0.18)	0.22 (0.17)	0.34 (0.17)
	BVSR	0.15 (0.03)	0.06 (0.01)	0.10 (0.08)	0.15 (0.07)	0.05 (0.06)
	BSLMM	0.41 (0.08)	0.34 (0.14)	0.22 (0.14)	0.26 (0.14)	0.29 (0.17)
PGE	BSLMM	0.12 (0.13)	0.21 (0.14)	0.27 (0.26)	0.46 (0.30)	0.18 (0.20)

PVE estimates are obtained using LMM, BVSR and BSLMM, while PGE estimates are obtained using BSLMM. Values in parentheses are standard error (for LMM) or standard deviation of posterior samples (for BVSR and BSLMM). 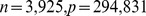 for height, and 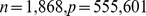 for other four traits.

These results, with LMM and BSLMM providing similar estimates of PVE, and estimates from BVSR being substantially lower, are consistent with simulation results for a trait with substantial polygenic component. One feature of BSLMM, not possessed by the other two methods, is that it can be used to attempt to quantify the relative contribution of a polygenic component, through estimation of PGE, which is the proportion of total genetic variance explained by “large” effect size SNPs (or more precisely, by the additional effects of those SNPs above a polygenic background). Since the PGE is defined within an inevitably over-simplistic model, specifically that effect sizes come from a mixture of two normal distributions, and also because it will be influenced by unmeasured environmental factors that correlate with genetic factors, we caution against over-interpreting the estimated values. We also note that estimates of PGE for these data (Table 2) are generally not very precise (high posterior standard deviation). Nonetheless, it is interesting that the estimated PGE for height, at 0.12, is lower than for any of the lipid traits (ranging from 0.18 for TG to 0.46 for TC), and that all these estimates suggest a substantial contribution from small polygenic effects in all five traits.

### Predicting Disease Risk in the WTCCC Dataset

To assess predictive performance on real data, we turn to the Wellcome trust case control consortium (WTCCC) 1 study [62], which have been previously used for assessing risk prediction [63]–[65]. These data include about 14,000 cases from seven common diseases and about 3,000 shared controls, typed at a total of about 450,000 SNPs. The seven common diseases are bipolar disorder (BD), coronary artery disease (CAD), Crohn's disease (CD), hypertension (HT), rheumatoid arthritis (RA), type 1 diabetes (T1D) and type 2 diabetes (T2D).

We compared the prediction performance of LMM, BVSR and BSLMM for all seven diseases. Following [64], we randomly split the data for each disease into a training set (80% of individuals) and a test set (remaining 20%), performing 20 such splits for each disease. We estimated parameters from the training set by treating the binary case control labels as quantitative traits, as in [5], [9]. [This approach can be justified by recognizing the linear model as a first order Taylor approximation to a generalized linear model; we discuss directly modeling binary phenotypes in the Discussion section.] We assess prediction performance in the test set by area under the curve (AUC) [66].

**Figure 3:**
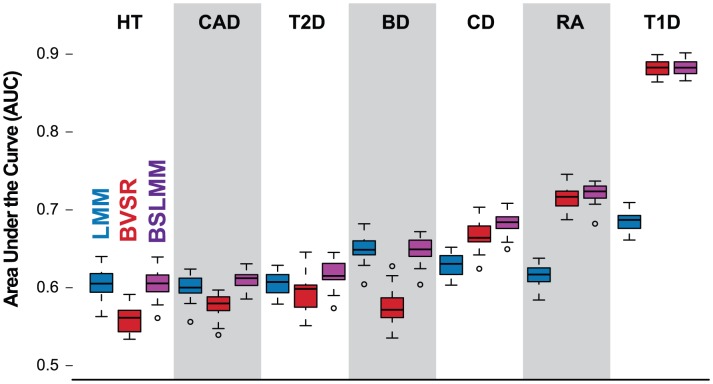
Comparison of prediction performance of LMM (blue), BVSR (red), and BSLMM (purple) for seven diseases in the WTCCC dataset. Performance is measured by area under the curve (AUC), where a higher value indicates better performance. The order of the diseases is based on the performance of BSLMM. The mean and standard deviation of AUC scores for BSLMM in the seven diseases are 0.60 (0.02) for HT, 0.60 (0.03) for CAD, 0.61 (0.03) for T2D, 0.65 (0.02) for BD, 0.68 (0.02) for CD, 0.72 (0.01) for RA, 0.88 (0.01) for T1D.

Figure 3 shows AUC for the three methods on all seven diseases. As in our simulations, we find BSLMM performs as well as or better than either of the other two methods for all seven diseases. Indeed, the performance of BSLMM appears to compare favorably with previous methods applied to the same dataset [63]–[65] (a precise comparison with previous results is difficult, as some studies use slightly different splitting strategies [63], [65] and some do not perform full cross validation [64]). As might be expected from the simulation results, BVSR performs better than LMM in diseases where a small number of relatively strong associations were identified in the original study [62] (CD, RA and T1D) and worse in the others. We obtained qualitatively similar results when we measured performance using the Brier score instead of AUC (Text S3; Figure S4).

Finally, we caution that, although BSLMM performs well here relative to other methods, at the present time, for these diseases, its prediction accuracy is unlikely to be of practical use in human clinical settings. In particular, in these simulations the number of cases and controls in the test set is roughly equal, which represents a much easier problem than clinical settings where disease prevalence is generally low even for common diseases (see [64] for a relevant discussion).

### Predicting Quantitative Phenotypes in Heterogeneous Stock Mice

In addition to the WTCCC dataset, we also assess perdition performance using a mouse dataset [67], which has been widely used to compare various phenotype prediction methods [37]–[39]. The mouse dataset is substantially smaller than the human data (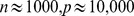, with exact numbers varying slightly depending on the phenotype and the split). This makes it computationally feasible to compare with a wider range of other methods. Therefore, we include in our comparisons here five other related approaches, some of which have been proposed previously for phenotype prediction. Specifically we compare with:

1. **LMM-Bayes**, a Bayesian version of the LMM, which we fit by setting 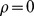 in BSLMM using our software.
2. **Bayesian Lasso** [39], [68], implemented in the R package BLR [39].
3. **BayesA-Flex**, our own modification of BayesA, which assumes a 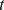 distribution for the effect sizes. Our modification involves estimating the scale parameter associated with the 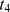 distribution from the data (Text S1). Although the original BayesA performs poorly in this dataset [38], this simple modification greatly improves its prediction performance (emphasizing again the general importance of estimating hyper-parameters from the data rather than fixing them to arbitrary values). We modified the R package BLR [39] to obtain posterior samples from this model.
4. **BayesC** 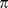 **,** implemented in the online software GenSel [34]. This implementation does not allow random effects, and therefore uses the same model as our BVSR, although with different prior distributions.
5. **BSLMM-EB** (where EB stands for empirical Bayes; this is a slight abuse of terminology, since in this method the estimated hyper parameters are obtained under the model with 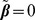), an approximation method to fit BSLMM. The method fixes the variance component 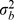 to its REML (restricted maximum likelihood) estimate obtained with 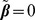, which is one of several strategies used in previous studies to alleviate the computational burden of fitting models similar to BSLMM [37], [41]. We sample posteriors from this model using our software.

See Text S1 for further details.

Following previous studies that have used these data for prediction [37]–[39] we focused on three quantitative phenotypes: CD8, MCH and BMI. These phenotypes have very different estimated narrow sense heritabilities: 0.89, 0.48, and 0.13 respectively [69]. Table S1 lists estimates of some key quantities for the three traits – including PVE, PGE and 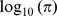 – obtained from LMM, BVSR and BSLMM. All three methods agree well on the PVE estimates, suggesting that the data is informative enough to overwhelm differences in prior specification for PVE estimation.

Following [37], [38], we divide the mouse dataset roughly half and half into a training set and a test set. As the mice come from 85 families, and individuals within a family are more closely related than individuals from different families, we also follow previous studies and use two different splits of the data: the *intra-family* split mixes all individuals together and randomly divides them into two sets of roughly equal size; the *inter-family* split randomly divides the 85 families into two sets, where each set contains roughly half of the individuals. We perform 20 replicates for each split of each phenotype. It is important to note that the intra-family split represents an easier setting for phenotype prediction, not only because individuals in the test set are more related genetically to those in the training set, but also because the individuals in the test set often share a similar environment with those in the training set (specifically, in the intra-family split, many individuals in the test set share a cage with individuals in the training set, but this is not the case in the inter-family split).

We apply each method using genotypes only, without other covariates. We obtain effect size estimates in the training dataset, and assess prediction performance using these estimates in the test set by root of mean square error (RMSE), where the mean is across individuals in the test set. We contrast the performance of other methods to BSLMM by calculating the RMSE difference, where a positive number indicates worse performance than BSLMM. We perform 20 inter-family splits and 20 intra-family splits for each phenotype.

**Figure 4:**
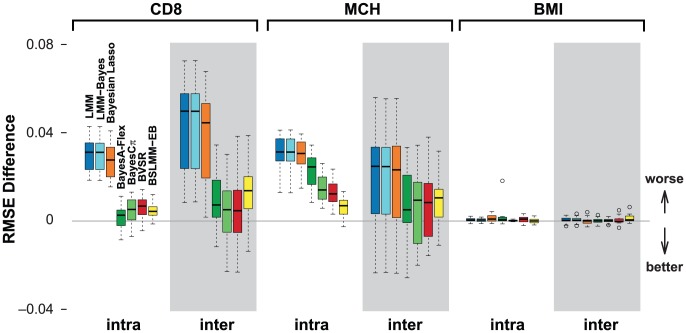
Comparison of prediction performance of several models with BSLMM for three traits in the heterogenous stock mouse dataset. Performance is measured by RMSE difference with respect to BSLMM, where a positive value indicates worse performance than BSLMM. The x-axis shows two different ways to split the data into a training set and a test set, each with 20 replicates. The mean RMSE of BSLMM for the six cases are 0.70, 0.80, 0.79, 0.90, 0.98 and 0.99, respectively.

Figure 4 summarizes the prediction accuracy, measured by RMSE, of each method compared against BSLMM. Measuring prediction performance by correlation gives similar results (Figure S5). For the low-heritability trait BMI, where no measured SNP has a large effect, all methods perform equally poorly. For both the more heritable traits, CD8 and MCH, BSLMM consistently outperformed all other methods, which seem to split into two groups: LMM, LMM-Bayes and Bayesian Lasso perform least well and similarly to one another on average; BVSR, BayesA-Flex, BayesC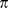 and BSLMM-EB perform better, and similarly to one another on average. A general trend here is that accuracy tends to increase as model assumptions improve in their ability to capture both larger genetic effects, and the combined “polygenic” contribution of smaller genetic effects (and possibly also confounding environmental effects correlating with genetic background). In particular, the 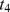 distribution underlying BayesA-Flex, which has a long tail that could capture large effects, performs noticeably better than either the normal or double-exponential distributions for effect sizes underlying LMM and Bayesian Lasso.

Comparisons of pairs of closely-related methods yield additional insights into factors that do and do not affect prediction accuracy. The fact that BSLMM performs better than BSLMM-EB illustrates how the approximation used in BSLMM-EB can degrade prediction accuracy, and thus demonstrates the practical benefits of our novel computational approach that avoids this approximation. Similarly, the superior performance of BayesA-Flex over BayesA (which performed poorly; not shown) also illustrates the benefits of estimating hyper parameters from the data, rather than fixing them to pre-specified values. The similar performance between BVSR and BayesC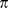, which fit the same model but with different priors, suggests that, for these data, results are relatively robust to prior specification. Presumably, this is because the data are sufficiently informative to overwhelm the differences in prior.

### Computational Speed

The average computational time taken for each method on the Mouse data is shown in Table 3. Some differences in computational time among methods may reflect implementational issues, including the language environment in which the methods are implemented, rather than fundamental differences between algorithms. In addition, computing times for many methods will be affected by the number of iterations used, and we did not undertake a comprehensive evaluation of how many iterations suffice for each algorithm. Nonetheless, the results indicate that our implementation of BSLMM is competitive in computing speed with the other (sampling-based) implementations considered here.

**Table 3:** Mean computation time, in hours, of various methods for the mouse dataset.

Method	Time in hrs (sd)
LMM	0.007 (0.002)
LMM-Bayes	0.14 (0.02)
BSLMM-EB	2.44 (3.52)
BSLMM	3.86 (4.13)
BVSR	9.20 (6.36)
BayesC 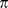	39.4 (11.7)
BayesA-Flex	68.6 (8.06)
Bayesian Lasso	78.6 (23.4)

Values in parentheses are standard deviations. Means and standard deviations are calculated based on 2.1 million MCMC iterations in 120 replicates: 20 intra-family and 20 inter-family splits for three phenotypes. Computations were performed on a single core of an Intel Xeon L5420 2.50 GHz CPU. Since computing times for many methods will vary with number of iterations used, and we did not undertake a comprehensive evaluation of how many iterations suffice for each algorithm, these results provide only a very rough guide to the relative computational burden of different methods.

In particular, we note that BSLMM is computationally faster than BVSR. This is unexpected, since BSLMM is effectively BVSR plus a random effects term, and the addition of a random effects term usually complicates computation. The explanation for this is that the (per-iteration) computational complexity of both BSLMM and BVSR depends, quadratically, on the number of selected SNPs in the sparse effects term (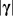), and this number can be substantially larger with BVSR than with BSLMM, because with BVSR additional SNPs are included to mimic the effect of the random effects term in BSLMM. The size of this effect will vary among datasets, but it can be substantial, particularly in cases where there are a large number of causal SNPs with small effects.

To illustrate this, Table 4 compares mean computation time for BSLMM vs BVSR for all datasets used here. For simulated data with a small number of causal SNPs, BSLMM and BVSR have similar computational times. However, in other cases (e.g. PVE = 0.6, S = 10,000 in Scenario I) BSLMM can be over an order of magnitude faster than BVSR. In a sense, this speed improvement of BSLMM over BVSR is consistent with its hybrid nature: in highly polygenic traits, BSLMM tends to behave similarly to LMM, resulting in a considerable speed gain.

**Table 4:** Mean computation time, in hours, of BVSR and BSLMM in all examples used in this study.

Simulations						
	PVE = 0.2, Number of Causal SNPs					
	10	100	1000	10000	10/10000	100/10000
BVSR	8.25 (1.11)	50.0 (17.3)	46.3 (25.0)	32.8 (25.0)	50.6 (23.9)	35.2 (24.7)
BSLMM	8.44 (2.04)	16.9 (4.06)	5.44 (0.23)	5.19 (0.20)	29.3 (17.2)	37.5 (19.1)
	PVE = 0.6, Number of Causal SNPs					
	10	100	1000	10000	10/10000	100/10000
BVSR	7.43 (0.72)	36.9 (5.71)	118 (8.94)	96.1 (12.0)	75.4 (28.9)	92.8 (15.0)
BSLMM	7.34 (1.05)	41.2 (5.12)	39.8 (15.0)	5.13 (0.34)	13.5 (5.52)	45.9 (16.6)

Real Data								
	Diseases							
	BD	CAD	CD	HT	RA	T1D	T2D	
BVSR	131 (20.3)	110 (20.3)	105 (13.9)	114 (21.0)	12.4 (3.52)	14.8 (2.47)	123 (22.0)	
BSLMM	21.7 (13.6)	22.0 (16.2)	57.6 (24.6)	32.8 (23.8)	9.58 (1.17)	14.5 (2.52)	38.7 (20.3)	
	Quantitative Traits							
	Height	HDL	LDL	TC	TG	CD8	MCH	BMI
BVSR	96.1	2.94	21.4	24.4	18.8	5.89 (1.87)	10.5(5.83)	11.2(8.30)
BSLMM	77.4	3.35	7.24	24.0	6.53	1.14 (1.05)	2.45(3.13)	7.97(3.79)

Computations were performed on a single core of an Intel Xeon L5420 2.50 GHz CPU, with 2.1 million MCMC iterations. Values in parentheses are standard deviations. Means and standard deviations are calculated based on 20 replicates in simulations, 20 replicates in the WTCCC dataset, and 40 replicates—20 intra-family and 20 inter-family splits—in the mouse dataset.

## Discussion

We have presented novel statistical and computational methods for BSLMM, a hybrid approach for polygenic modeling for GWAS data that simultaneously allows for both a small number of individually large genetic effects, and combined effects of many small genetic effects, with the balance between the two being inferred from the data in hand. This hybrid approach is both computationally tractable for moderately large datasets (our implementation can handle at least 10,000 individuals with 500,000 SNPs on our moderately-equipped modern desktop computer), and is sufficiently flexible to perform well in a wide range of settings. In particular, depending on the genetic architecture, BSLMM is either as accurate, or more accurate, than the widely-used LMM for estimating PVE of quantitative traits. And for phenotype prediction BSLMM consistently outperformed a range of other approaches on the examples we considered here. By generalizing two widely-used models, and including both as special cases, BSLMM should have many applications beyond polygenic modeling. Indeed, despite its increased computational burden, we believe that BSLMM represents an attractive alternative to the widely-used LASSO [70] for general regression-based prediction problems.

Although it was not our focus here, BSLMM can be easily modified to analyze binary phenotypes, including for example, a typical human case-control GWAS. For PVE estimation, one can directly apply BSLMM, treating the 1/0 case-control status as a quantitative outcome, and then apply a correction factor derived by [24] to transform this estimated PVE on the “observed scale” to an estimated PVE on a latent liability scale. This correction, for which we supply an alternative derivation in Text S3, corrects for both ascertainment and the binary nature of case-control data. For phenotype prediction, one can again directly apply BSLMM, treating the 1/0 case-control status as a quantitative outcome, as we do here for the WTCCC dataset, and interpret the resulting phenotype predictions as the estimated probability of being a case. Although in principle one might hope to improve on this by modifying BSLMM to directly model the binary outcomes, using a probit link for example, we have implemented this probit approach and found that not only is it substantially more computationally expensive (quadratic in 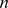 instead of linear in 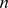), but it performed slightly worse than treating the binary outcomes as quantitative, at least in experiments based on the mouse phenotypes considered here (Text S3 and Figure S6), which is consistent with previous findings in quantitative trait loci mapping [71]. This may partly reflect inaccuracies introduced by the known greater computational burden and corresponding mixing issues with probit models (e.g. [72]) which are magnified here by the polygenic nature of the traits, and partly reflect robustness of linear models to model misspecification.

The computational innovations we introduce here, building on work by [8], [9], make BSLMM considerably more tractable than it would otherwise be. Nonetheless, the computational burden, as with other posterior sampling based methods, remains heavy, both due to memory requirements (e.g. to store all genotypes) and CPU time (e.g. for the large number of sampling iterations required for reasonable convergence). Although more substantial computational resources will somewhat increase the size of data that can be tackled, further methodological innovation will likely be required to apply BSLMM to the very large datasets that are currently being collected.

In addition to providing a specific implementation that allows BSLMM to be fitted to moderately large datasets, we hope that our work also helps highlight some more general principles for improving polygenic modeling methodology. These include:

1. The benefits of characterizing different methods by their effect size distribution assumptions. While this point may seem obvious, and is certainly not new (e.g. [40], [73]), polygenic modeling applications often focus on the algorithm used to fit the model, rather than the effect size distribution used. While computational issues are very important, and often interact with modeling assumptions, we believe it is important to distinguish, conceptually, between the two. One benefit of characterizing methods by their modeling assumptions is that it becomes easier to predict which methods will tend to do well in which settings.
2. The importance of selecting a sufficiently flexible distribution for effect sizes. The version of BSLMM we focus on here (with 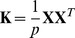) assumes a mixture of two (zero-mean) normals for the effect size distribution. Our examples demonstrate the gain in performance this achieves compared to less flexible distributions such as a single normal (LMM) or a point-normal (BVSR). More generally, in our phenotype prediction experiments, methods with more flexible effect size distributions tended to perform better than those with less flexible distributions.
3. The importance of estimating hyper-parameters from data, rather than fixing them to pre-specified values. Here we are echo-ing and reinforcing similar themes emphasized by [34] and [19]. Indeed, our comparison between BSLMM and BSLMM-EB for phenotype prediction illustrates the benefits not only of estimating hyper-parameters from the data, but of doing so in an integrated way, rather than as a two-step procedure.
4. The attractiveness of specifying prior distributions for hyper-parameters by focusing on the proportion of variance in phenotypes explained by different genetic components (e.g. PVE and PGE in our notation). This idea is not limited to BSLMM, and could be helpful even with methods that make use of other effect size distributions.

One question to which we do not know the answer is how often the mixture of two normal distributions underlying BSLMM will be sufficiently flexible to capture the actual effect size distribution, and to what extent more flexible distributional assumptions (e.g. a mixture of more than two normals, or a mixture of 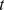 distributions with degrees of freedom estimated from the data) will produce meaningful gains in performance. It seems likely that, at least in some cases, use of a more flexible distribution will improve performance, and would therefore be preferable if it could be accomplished with reasonable computational expense. Unfortunately some of the tricks we use to accomplish computational gains here may be less effective, or difficult to apply, for more flexible distributions. In particular, the tricks we use from [8] and [9] may be difficult to extend to allow for mixtures with more than two components. In addition, for some choices of effect size distribution, one might have to perform MCMC sampling on the effect sizes 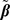 directly, rather than sampling 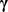, integrating 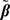 out analytically, as we do here. It is unclear whether this will necessarily result in a loss of computational efficiency: sampling 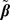 reduces computational expense per update at the cost of increasing the number of updates necessary (sampling 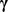 by integrating over 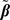 analytically ensures faster mixing and convergence [74], [75]). Because of these issues, it is difficult to predict which effect size distributions will ultimately provide the best balance between modeling accuracy and computational burden. Nonetheless, compared with currently available alternatives, we believe that BSLMM strikes an appealing balance between flexibility, performance, and computational tractability.

## Supporting Information

Figure S1 Comparison of prediction performance of LMM (blue), BVSR (red) and BSLMM (purple) in two simulation scenarios, where all causal SNPs are excluded from the data. Performance is measured by Relative Predictive Gain (RPG). True PVE = 0.6. Means and standard deviations (error bars) are based on 20 replicates. The x-axis show the number of causal SNPs (Scenario I) or the number of medium/small effect SNPs (Scenario II).
(EPS)

Figure S2 Comparison of prediction performance of LMM (blue), BVSR (red) and BSLMM (purple) in two simulation scenarios, where all causal SNPs are included in the data. Performance is measured by correlation. True PVE = 0.6. Means and standard deviations (error bars) are based on 20 replicates. The x-axis show the number of causal SNPs (Scenario I) or the number of medium/small effect SNPs (Scenario II).
(EPS)

Figure S3 Comparison of prediction performance of LMM (blue), BVSR (red) and BSLMM (purple) in two simulation scenarios, where all causal SNPs are excluded from the data. Performance is measured by correlation. True PVE = 0.6. Means and standard deviations (error bars) are based on 20 replicates. The x-axis show the number of causal SNPs (Scenario I) or the number of medium/small effect SNPs (Scenario II).
(EPS)

Figure S4 Comparison of prediction performance of LMM (blue), BVSR (red) and BSLMM (purple) for seven diseases in the WTCCC dataset. Performance is measured by Brier score, where a lower score indicates better performance. The order of the diseases is based on the performance of BSLMM. The mean and standard deviation of Brier scores for BSLMM in the seven diseases are 0.232 (0.004) for HT, 0.230 (0.004) for CAD, 0.230 (0.003) for T2D, 0.222 (0.004) for BD, 0.211 (0.004) for CD, 0.204 (0.004) for RA, 0.139 (0.006) for T1D.
(EPS)

Figure S5 Comparison of prediction performance of several models with BSLMM for three traits in the heterogenous stock mouse dataset. Performance is measured by correlation difference with respect to BSLMM, where a positive value indicates better performance than BSLMM. The x-axis shows two different ways to split the data into a training set and a test set, each with 20 replicates. The mean correlation of BSLMM for the six cases are 0.72, 0.61, 0.61, 0.47, 0.21 and 0.14, respectively.
(EPS)

Figure S6 Comparison of prediction performance between BSLMM and probit BSLMM for three binary traits in the heterogenous stock mouse dataset. Performance is measured by Brier score difference with respect to BSLMM, where a positive value indicates worse performance than BSLMM. The x-axis shows two different ways to split the data into a training set and a test set, each with 20 replicates. The mean Brier scores of BSLMM for the six cases are 0.185, 0.205, 0.201, 0.236, 0.245 and 0.249, respectively.
(EPS)

Table S1Estimates of PVE, PGE and 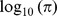 for CD8, MCH and BMI in the mouse dataset. PVE estimates are obtained using LMM, BVSR and BSLMM, 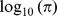 estimates are obtained using BVSR and BSLMM, and PGE estimates are obtained using BSLMM. Values in parentheses are standard error (for LMM) or standard deviation of posterior samples (for BVSR and BSLMM). 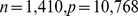 for CD8, 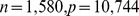 for MCH, and 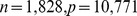 for BMI.(PDF)

Text S1 Detailed Methods.
(PDF)

Text S2 Detailed MCMC Strategy for BSLMM.
(PDF)

Text S3 The Probit BSLMM and Binary Traits.
(PDF)
