# Supplementary material for: Polygenic Modeling with Bayesian Sparse Linear Mixed Models
Source: PLoS Genet. 2013 Feb 7;9(2):e1003264. doi: 10.1371/journal.pgen.1003264 (PMC3567190; doi:10.1371/journal.pgen.1003264)
Supplement: Table S1 — Estimates of PVE, PGE and for CD8, MCH and BMI in the mouse dataset. PVE estimates are obtained using LMM, BVSR and BSLMM, estimates are obtained using BVSR and BSLMM, and PGE estimates are obtained using BSLMM. Values in parentheses are standard error (for LMM) or standard deviation of posterior samples (for BVSR and BSLMM). for CD8, for MCH, and for BMI. (PDF) [file pgen.1003264.s007.pdf]

Table S1. Estimates of PVE, PGE and $\log_{10}(\pi)$ for CD8, MCH and BMI in the mouse data set. PVE estimates are obtained using LMM, BVSR and BSLMM, $\log_{10}(\pi)$ estimates are obtained using BVSR and BSLMM, and PGE estimates are obtained using BSLMM. Values in parentheses are standard error (for LMM) or standard deviation of posterior samples (for BVSR and BSLMM). $n = 1,410, p = 10,768$ for CD8, $n = 1,580, p = 10,744$ for MCH, and $n = 1,828, p = 10,771$ for BMI.

	Method	CD8	MCH	BMI
PVE	LMM	0.61 (0.03)	0.64 (0.03)	0.14 (0.03)
	BVSR	0.66 (0.02)	0.57 (0.02)	0.13 (0.02)
	BSLMM	0.64 (0.02)	0.61 (0.03)	0.13 (0.02)
PGE	BSLMM	0.50 (0.09)	0.43 (0.06)	0.40 (0.28)
$\log_{10}(\pi)$	BVSR	-1.69 (0.12)	-1.62 (0.09)	-1.52 (0.21)
	BSLMM	-2.68 (0.24)	-2.77 (0.19)	-2.44 (0.70)
