# Supplementary material for: Polygenic Modeling with Bayesian Sparse Linear Mixed Models
Source: PLoS Genet. 2013 Feb 7;9(2):e1003264. doi: 10.1371/journal.pgen.1003264 (PMC3567190; doi:10.1371/journal.pgen.1003264)
Supplement: Text S1 — Detailed Methods. (PDF) [file pgen.1003264.s008.pdf]

Text S1 Detailed Methods

GWAS Datasets

We used four GWAS data sets in the present study.

The first data set contains height measurements for 3925 Australian individuals [1]. We used the data set for simulation and PVE estimation. The phenotypes were already regressed out of age and sex effects, and were quantile normalized to a standard normal distribution afterwards [1]. A total of 294,831 SNPs were available after stringent quality control [1]. We imputed missing SNPs using IMPUTE2 [2], and further excluded five SNPs that have minor allele frequencies below 1% after imputation.

The second data set consists of blood lipid measurements for 1868 individuals from the Pharmacogenomics and Risk of Cardiovascular Disease (PARC) study, with experimental design and genotyping procedures detailed in [3]. We used this data for PVE estimation. The individuals came from two study groups: the Cholesterol and Pharmacogenetics (CAP) group [4] and the Pravastatin Inflammation/CRP Evaluation (PRINCE) group [5]. The PRINCE study consists of two cohorts: one contains individuals with history of cardiovascular diseases (CVD) and the other contains individuals with no history of CVD. From both study groups, we selected all 1868 individuals that have complete low-density lipoprotein (LDL) subfraction measurements. We selected four different blood lipid measurements as phenotypes in the present study: LDL, high-density lipoprotein (HDL), total cholesterol (TC) and triglycerides (TG). Phenotypes were quantile-normalized to a standard normal distribution within each group, corrected for covariates including BMI (body mass index), age, sex, and smoking status effects, and quantile-normalized again [3]. Individuals were typed on two different SNP arrays (Illumina HumanHap300 and HumanQuad610 bead chips, Illumina, San Diego, CA). We used all SNPs that appeared in either of the arrays and we imputed missing genotypes using IMPUTE2 [2]. We obtained a total of 582,962 SNPs and we used 555,601 polymorphic SNPs with minor allele frequency above 1% for analysis.

The third data set is from the Wellcome trust case control consortium (WTCCC) 1 study [6]. We used this data set to assess phenotype prediction performance. The data set consists of about 14,000 cases of seven common diseases, including 1868 cases of bipolar disorder (BD), 1926 cases of coronary artery disease (CAD), 1748 cases of Crohn's disease (CD), 1952 cases of hypertension (HT), 1860 cases rheumatoid arthritis (RA), 1963 cases of type 1 diabetes (T1D) and 1924 cases of type 2 diabetes (T2D), as well as 2938 shared controls. We obtained quality controlled genotypes from WTCCC and we further imputed missing genotypes using BIMBAM [7], which resulted in a total of 458,868 shared SNPs. All polymorphic SNPs with minor allele frequency above 1% in the training data were used for prediction (about 400,000 SNPs; depending on the disease and the split).

The fourth data set comes from a genetically heterogeneous stock of mice, consisting of 1904 individuals from 85 families, all descended from eight inbred progenitor strains [8]. We used this data set to assess phenotype prediction performance of several methods. Multiple phenotype measurements are available for the data set, and we selected three phenotypes among them: percentage of CD8+ cells (CD8, $n = 1410$), mean corpuscular hemoglobin (MCH, $n = 1580$) and body mass index (BMI, $n = 1828$). We selected these phenotypes because they were previously used for comparing prediction performance of various methods [9–11], and they represent a wide range of narrow sense heritability: CD8 has a high heritability, MCH has a median heritability and BMI has a low heritability [12]. All phenotypes were already corrected for sex, age, body weight, season and year effects [8], and we further quantile normalized the phenotypes to a standard normal distribution. A total of 12,226 autosomal SNPs were available for all mice. For individuals with missing genotypes, we imputed missing values by the mean genotype of that SNP in their family. All polymorphic SNPs with minor allele frequency above 1% in the training data were used for prediction (about 10,000 SNPs; depending on the phenotype and the split).

Simulations

We used genotypes from the human height data set [1] described above and simulated phenotypes from the simple linear model (1) with different assumptions for the distribution of effect sizes $\boldsymbol{\beta}$. We consider two simulation scenarios where the true PVE is known and we simulated 20 independent sets of phenotype data in each case.

Scenario I: the effect sizes of causal SNPs come from a normal distribution. We randomly chose a fixed number of causal SNPs (10, 100, 1000, 10000) and simulated their effect sizes from a $\mathrm{N}(0, 1)$ distribution. We drew the errors from a normal distribution with variance chosen to achieve a given PVE (0.2 and 0.6).

Scenario II: the effect sizes of causal SNPs come from a mixture of two normal distributions, such that a small group of causal SNPs have additional effects. We first randomly chose a large number of causal SNPs (10000), and among them, we further selected a small number of medium effect size SNPs (10 or 100) and used what were left as small effect size SNPs (9990 or 9900). We simulated the small effect sizes for all causal SNPs (10000) from a $\mathrm{N}(0, 1)$ distribution. Afterwards, we drew additional effect sizes (in addition to the small effects already drawn) for those medium effect SNPs (10 or 100) from a $\mathrm{N}(0, 1)$ distribution, and scaled these additional effect sizes further so that together they explained a fixed proportion of genetic variance, or PGE (0.1 and 0.2, for 10 and 100 medium effect size SNPs, respectively). Once we obtained the final effect sizes for all causal SNPs, we drew errors to achieve a given PVE (0.2 and 0.6).

Assessing Prediction Performance in Simulations

MSPE and RPG

We assess prediction accuracy mainly using mean square prediction error (MSPE), and a rescaled version of MSPE called relative predictive gain (RPG). The MSPE for predicting a future observation in the simple linear model (1) depends on comparing an estimated value of $\boldsymbol{\beta}$, $\hat{\boldsymbol{\beta}}$, the true value of $\boldsymbol{\beta}$, and the error variance τ, as follows:

$$\mathrm{MSPE}(\hat{\boldsymbol{\beta}}; \boldsymbol{\beta}, \tau) := E(\mathbf{x}^T \hat{\boldsymbol{\beta}} - y)^2 \tag{24}$$

$$= E((\sum_{i=1}^{p} x_i(\hat{\beta}_i - \beta_i))^2) + \tau^{-1} \tag{25}$$

$$= \sum_{i=1}^{p} \sum_{j=1}^{p} r_{ij}(\hat{\beta}_i - \beta_i)(\hat{\beta}_j - \beta_j) + \tau^{-1} \tag{26}$$

$$\approx \sum_{|i-j| \leq 20} s_{ij}(\hat{\beta}_i - \beta_i)(\hat{\beta}_j - \beta_j) + \tau^{-1}, \tag{27}$$

where y is the phenotype for a future observation, $\mathbf{x}$ is the corresponding p-vector of genotypes, $r_{ij} = E(x_i x_j)$ is the covariance between SNP i and j. In practice, we approximate r_{ij} with the sample covariance $s_{ij} = \frac{1}{n} \sum_{k=1}^{n} x_{ik} x_{jk}$, and we only consider s_{ij} for neighboring SNPs that satisfy $|i - j| \leq 20$. This is because linkage dis-equilibrium (LD) decays with distance and remote SNPs are approximately independent with each other. The above definition of MSPE extends the definition in [13] to take into account correlations among neighboring SNPs.

We denote MSPE_0 as the MSPE obtained using only the mean of the phenotype (i.e. $\bar{\mathbf{y}}$) for prediction, and we define RPG as the rescaled version of MSPE following [13]:

$$\mathrm{RPG}(\boldsymbol{\beta}, \hat{\boldsymbol{\beta}}) := \frac{\mathrm{MSPE}_0 - \mathrm{MSPE}(\hat{\boldsymbol{\beta}}; \boldsymbol{\beta}, \tau)}{\mathrm{MSPE}_0 - \mathrm{MSPE}(\boldsymbol{\beta}; \boldsymbol{\beta}, \tau)}. \tag{28}$$

When applying these formulae for LMM or BSLMM note that we use estimates $\hat{\boldsymbol{\beta}}$ for $\boldsymbol{\beta}$ in (1), and not for $\tilde{\boldsymbol{\beta}}$ in (6). This ensures that the resulting predictions take account of both the sparse effects $\boldsymbol{\beta}$ and the random effect $\mathbf{u}$ in (6). The way we obtain these estimates $\hat{\boldsymbol{\beta}}$ is detailed below.

Correlation

We also assess prediction accuracy using correlation. The correlation between the predicted value and the true value for a future observation in the simple linear model (1) depends on $\hat{\boldsymbol{\beta}}$, $\boldsymbol{\beta}$ and τ as follows:

$$\mathrm{Cor}(\hat{\boldsymbol{\beta}};\boldsymbol{\beta},\tau) := \mathrm{Cor}(\mathbf{x}^T\hat{\boldsymbol{\beta}}, y) \tag{29}$$

$$= \frac{E((\sum_{i=1}^p x_i\hat{\beta}_i)(\sum_{j=1}^p x_j\beta_j))}{\sqrt{E((\sum_{i=1}^p x_i\hat{\beta}_i)^2)E((\sum_{j=1}^p x_j\beta_j)^2)/\mathrm{PVE}(\beta,\tau)}} \tag{30}$$

$$= \frac{(\sum_{i=1}^p\sum_{j=1}^p r_{ij}\hat{\beta}_i\beta_j)\sqrt{\mathrm{PVE}(\beta,\tau)}}{\sqrt{(\sum_{i=1}^p\sum_{j=1}^p r_{ij}\hat{\beta}_i\hat{\beta}_j)(\sum_{i=1}^p\sum_{j=1}^p r_{ij}\beta_i\beta_j)}} \tag{31}$$

$$\approx \frac{(\sum_{|i-j|\leq 20} s_{ij}\hat{\beta}_i\beta_j)\sqrt{\mathrm{PVE}(\beta,\tau)}}{\sqrt{(\sum_{|i-j|\leq 20} s_{ij}\hat{\beta}_i\hat{\beta}_j)(\sum_{|i-j|\leq 20} s_{ij}\beta_i\beta_j)}}. \tag{32}$$

Again, when applying these formulae for LMM or BSLMM note that we use estimates $\hat{\boldsymbol{\beta}}$ for $\boldsymbol{\beta}$ in (1), and not for $\tilde{\boldsymbol{\beta}}$ in (6).

Details of BSLMM

Centering X and K

We assume that the genotypes in $\mathbf{X}$ have been measured on bi-allelic markers, and that the genotypes at each marker are coded as 0, 1 or 2 copies of some reference allele. (For imputed genotypes we use the posterior mean genotype [7].) It occasionally simplifies the algebra to assume that each column of $\mathbf{X}$ is centered to have mean 0; since the results will be the same with or without centering, we make this assumption throughout. It is also common to standardize the columns of $\mathbf{X}$ to have unit variance. In contrast to centering, standardizing the columns will affect the results, and we do not standardize the columns in our applications here, although all our methods could be applied with the matrix standardized in this way. (Standardizing the columns of $\mathbf{X}$ corresponds to making an assumption that rarer variants tend to have larger effects than common variants, and precisely that marker effect sizes tend to decay with the inverse of the genotype variance; see [14, 15] for relevant discussion.) In summary, throughout this paper $X_{ij} = (x_{ij} - \bar{x}_j)$ where x_{ij} is the number of copies of the reference allele at marker j in individual i, and $\bar{x}_j := (1/n)\sum_i x_{ij}$.

To facilitate prior specification, in addition to centering the genotype matrix $\mathbf{X}$, we also assume that the relatedness matrix $\mathbf{K}$ is "centered", in the sense that the random effects have mean zero: $\sum_{i=1}^n u_i = 0$. This holds automatically for $\mathbf{K} \propto \mathbf{X}\mathbf{X}^T$, with $\mathbf{X}$ centered. More generally it can be achieved by multiplying the relatedness matrix with a projection matrix on both sides: $\mathbf{MKM}$, where $\mathbf{M} = \mathbf{I}_n - \mathbf{1}_n(\mathbf{1}_n^T\mathbf{1}_n)^{-1}\mathbf{1}_n^T$. The resulting transformed relatedness matrix is positive-semidefinite as long as the original relatedness matrix is positive-semidefinite.

Definition and derivation of expressions for h and ρ

We define h, ρ

$$h(\pi, \sigma_a, \sigma_b) := \frac{E(\mathrm{V}(\mathbf{X}\tilde{\boldsymbol{\beta}} + \mathbf{u}))}{E(\mathrm{V}(\mathbf{X}\tilde{\boldsymbol{\beta}} + \mathbf{u})) + \tau^{-1}}, \tag{33}$$

$$\rho(\pi, \sigma_a, \sigma_b) := \frac{E(\mathrm{V}(\mathbf{X}\tilde{\boldsymbol{\beta}}))}{E(\mathrm{V}(\mathbf{X}\tilde{\boldsymbol{\beta}} + \mathbf{u}))}, \tag{34}$$

where the function $V(\mathbf{x})$ is defined in equation 15, and the expectations are taken with respect to $(\tilde{\boldsymbol{\beta}}, \mathbf{u})$, conditional on hyper parameters $(\sigma_a, \sigma_b, \pi, \tau)$. These conditional expectations are extensions of, and slight simplifications of, the similar expression for h in [13]; the simplification comes from taking expectations conditional on π instead of conditional on $\boldsymbol{\gamma}$. These definitions of h and ρ are motivated by approximating the expectations of PVE and PGE by the ratios of the expectations of the numerator and denominator. Both h and ρ take values between 0 and 1 and serve as rough guides to the expectations of PVE and PGE, respectively.

The expectations in the above expressions, conditional on hyper-parameters $(\sigma_a^2, \sigma_b^2, \pi, \tau)$, can be obtained as:

$$\mathrm{E}(\mathrm{V}(\mathbf{X}\tilde{\boldsymbol{\beta}})|\sigma_a^2, \pi, \tau^{-1}) = \mathrm{E}(\sum_{i=1}^{p} \mathrm{V}(\mathbf{x}_i\tilde{\beta}_i)|\sigma_a^2, \pi, \tau^{-1}) = p\pi s_a \sigma_a^2 \tau^{-1}, \tag{35}$$

$$\mathrm{E}(\mathrm{V}(\mathbf{X}\tilde{\boldsymbol{\beta}} + \mathbf{u})|\sigma_a^2, \sigma_b^2, \pi, \tau^{-1}) = \mathrm{E}(\sum_{i=1}^{p} \mathrm{V}(\mathbf{x}_i\tilde{\beta}_i) + \mathrm{V}(\mathbf{u})|\sigma_a^2, \sigma_b^2, \pi, \tau^{-1}) = p\pi s_a \sigma_a^2 \tau^{-1} + s_b \sigma_b^2 \tau^{-1}, \tag{36}$$

where $\mathbf{x}_i$ is the ith column of $\mathbf{X}$, $s_a = \frac{1}{np}\sum_{i=1}^{p}\sum_{j=1}^{n} x_{ij}^2$, $s_b = \frac{1}{n}\sum_{i=1}^{n} k_{ii}$, x_{ij} and k_{ij} are the ijth elements of matrices $\mathbf{X}$ and $\mathbf{K}$, respectively. The above derivation assumes centered genotypes and relatedness matrix.

Plugging these approximations into the expressions (13) and (14) gives (16) and (17).

Induced Priors on σ_a^2 and σ_b^2

Solving (16) and (17) for σ_a^2 and σ_b^2 as functions of h, ρ and π gives:

$$\sigma_a^2 = \frac{h\rho}{(1-h)p\pi s_a}, \tag{37}$$

$$\sigma_b^2 = \frac{h(1-\rho)}{(1-h)s_b}. \tag{38}$$

The independent priors (18), (19), (12) for (h, ρ, π) induce a joint prior on $(\sigma_a, \sigma_b, \pi)$:

$$p(\sigma_a^2, \sigma_b^2, \pi) \propto \frac{p s_a s_b}{(p\pi s_a \sigma_a^2 + s_b \sigma_b^2 + 1)^2 (p\pi s_a \sigma_a^2 + s_b \sigma_b^2)}, \tag{39}$$

which is heavy tailed for (σ_a^2, σ_b^2) marginally (i.e. tail has a polynomial decay), a feature desirable in association studies [15]. The priors also have another nice property that the marker effect size variance σ_a^2 tends to decrease as the proportion of markers with an effect (π) increases.

PVE Estimation with LMM, BVSR and BSLMM

We could estimate PVE using the posterior mean of the MCMC samples for all three models, and we do so for both BVSR and BSLMM. For LMM, we follow previous studies [1] and use an approximation to

the PVE. In particular, we consider the LMM defined by equations (6)-(7) with $\tilde{\boldsymbol{\beta}} = 0$, and we estimate PVE by the ratio of expectations

$$\hat{\text{PVE}} = \frac{s_b \hat{\sigma}_b^2}{s_b \hat{\sigma}_b^2 + 1}, \tag{40}$$

where $\hat{\sigma}_b^2$ is the REML estimate for the variance component. This formula can be viewed as a generalization of the form used in [1], and is valid for any choice of centered relatedness matrix $\mathbf{K}$.

To obtain the standard error of the above estimate, we compute the second derivative of the log restricted likelihood function with respect to σ_b^2 [16] and evaluate it at $\hat{\sigma}_b^2$:

$$l_r''(\hat{\sigma}_b^2) = \frac{\partial l_r^2}{\partial^2 \sigma_b^2}|_{\sigma_b^2 = \hat{\sigma}_b^2} = \frac{1}{2}\text{trace}(\mathbf{PKPK}) - \frac{n-2}{2}\frac{2(\mathbf{y}^T\mathbf{Py})(\mathbf{y}^T\mathbf{Py}) - (\mathbf{y}^T\mathbf{Py})^2}{\mathbf{y}^T\mathbf{Py}}|_{\sigma_b^2 = \hat{\sigma}_b^2}, \tag{41}$$

where l_r denotes the log restricted likelihood, $\mathbf{H} = \sigma_b^2\mathbf{K} + \mathbf{I}_n$ and $\mathbf{P} = \mathbf{H}^{-1} - \mathbf{H}^{-1}\mathbf{1}_n(\mathbf{1}_n\mathbf{H}^{-1}\mathbf{1}_n^T)^{-1}\mathbf{1}_n^T\mathbf{H}^{-1}$. Despite its complicated form, the second derivative can be easily and efficiently evaluated using recursions in [16]. As the variance of $\hat{\sigma}_b^2$ is asymptotically $\text{v}(\hat{\sigma}_b^2) = -1/l''(\hat{\sigma}_b^2)$, using the delta method, we approximate the standard error of $\hat{\text{PVE}}$ by

$$\text{se}(\hat{\text{PVE}}) \approx \frac{s_b}{(s_b \hat{\sigma}_b^2 + 1)^2}\sqrt{\text{v}(\hat{\sigma}_b^2)}. \tag{42}$$

As a check on correctness, we also used the posterior mean for PVE obtained from LMM-Bayes to estimate PVE. This gave almost identical results in all cases considered here and therefore only results from LMM are presented.

Phenotype Prediction with LMM, BVSR and BSLMM

Real Data

In the real data, we can perform prediction by estimating both the sparse effects $(\mu, \tilde{\boldsymbol{\beta}})$ and the random effects $(\mathbf{u})$ in (6) from the training set, and use these to predict phenotypes in the test set.

Let $\hat{\mu}_o$, $\hat{\tilde{\boldsymbol{\beta}}}_o$ and $\hat{\mathbf{u}}_o$ denote estimates for the sparse and random effects obtained from the observed (training) sample. For BSLMM and BVSR these estimates are the posterior means for these parameters, estimated from MCMC samples (for BVSR $\hat{\mathbf{u}}_o \equiv 0$). For LMM $\hat{\mathbf{u}}_o$ is obtained as the conditional posterior mean of $\mathbf{u}_o$ given the REML estimate for σ_b^2 (i.e. BLUP).

We then obtain predictions for a future (test) sample as follows. For a general relatedness matrix $\mathbf{K}$, we assume that the random effects for the observed and future samples follow a multivariate normal distribution

$$\begin{pmatrix} \mathbf{u}_o \\ \mathbf{u}_f \end{pmatrix} \sim \text{MVN}_{n_o+n_f}(\begin{pmatrix} 0 \\ 0 \end{pmatrix}, \begin{pmatrix} \mathbf{K}_{oo} & \mathbf{K}_{of} \\ \mathbf{K}_{fo} & \mathbf{K}_{ff} \end{pmatrix}), \tag{43}$$

where n_o and n_f are the sample size for observed (training) and future (test) data, respectively, and the joint covariance matrix is centered based on training data. Standard multivariate normal theory gives the conditional distribution

$$\mathbf{u}_f|\mathbf{u}_o \sim \text{MVN}_{n_f}(\mathbf{K}_{fo}\mathbf{K}_{oo}^{-1}\mathbf{u}_o, \mathbf{K}_{ff} - \mathbf{K}_{fo}\mathbf{K}_{oo}^{-1}\mathbf{K}_{of}). \tag{44}$$

We use the conditional mean as an estimate for $\mathbf{u}_f$ and thus the predicted phenotypes for future observations are

$$\hat{\mathbf{y}}_f = \mathbf{1}_{n_f}\hat{\mu}_o + \mathbf{X}_f\hat{\tilde{\boldsymbol{\beta}}}_o + \mathbf{K}_{fo}\mathbf{K}_{oo}^{-1}\hat{\mathbf{u}}_o, \tag{45}$$

where $\mathbf{X}_f$ is the genotype matrix for the test data, with each column centered using the mean from the training data.

Simulated Data

In the simulated data we use RPG and correlation to assess prediction accuracy. To compute RPG and correlation we need to obtain estimates for $\boldsymbol{\beta}$ in the simple linear model (1). To do this, in the special case when the relatedness matrix $\mathbf{K} = \frac{1}{p}\mathbf{X}\mathbf{X}^T$, we rewrite the model (6) as

$$\mathbf{y} = \mathbf{1}_n\mu + \mathbf{X}\tilde{\boldsymbol{\beta}} + \mathbf{X}\boldsymbol{\alpha} + \boldsymbol{\epsilon}, \tag{46}$$

$$\boldsymbol{\epsilon} \sim \text{MVN}_n(0, \tau^{-1}\mathbf{I}_n), \tag{47}$$

$$\tilde{\beta}_i \sim \pi\text{N}(0, \sigma_a^2\tau^{-1}) + (1-\pi)\delta_0, \tag{48}$$

$$\alpha_i \sim \text{N}(0, \sigma_b^2/(p\tau)), \tag{49}$$

where we can think of the p-vector $\boldsymbol{\alpha}$ as representing the "small" effect sizes present at every locus. The special case of $\pi = 0$ ($\tilde{\boldsymbol{\beta}} \equiv 0$) gives LMM, and the special case of $\sigma_b^2 = 0$ ($\boldsymbol{\alpha} \equiv 0$) gives BVSR. Note that $\boldsymbol{\alpha} + \tilde{\boldsymbol{\beta}} = \boldsymbol{\beta}$, so summing estimates of $\boldsymbol{\alpha}$ and $\tilde{\boldsymbol{\beta}}$ yields an estimate for $\boldsymbol{\beta}$ in (1).

For LMM we estimate $\boldsymbol{\alpha}$ by its conditional expectation

$$\hat{\boldsymbol{\alpha}} = \frac{\hat{\sigma}_b^2}{p}\mathbf{X}^T(\hat{\sigma}_b^2\mathbf{K} + \mathbf{I}_n)^{-1}\mathbf{y}, \tag{50}$$

where $\hat{\sigma}_b^2$ is the REML estimate of the variance component in the observed data. Since $\tilde{\boldsymbol{\beta}} \equiv 0$ in LMM, this estimate for $\boldsymbol{\alpha}$ provides the required estimate for $\boldsymbol{\beta}$ in (1).

For BVSR, we use the posterior mean of $\tilde{\boldsymbol{\beta}}$ (since $\boldsymbol{\alpha} \equiv 0$).

For BSLMM, we use Rao-Blackwellisation to obtain an approximation to the posterior mean for $\boldsymbol{\alpha}$ (Text S2), and then add this to the (approximate) posterior mean for $\tilde{\boldsymbol{\beta}}$ obtained from the MCMC sampler to obtain an approximation for the posterior mean of $\boldsymbol{\beta}$ in (1).

Other Methods

1. **LMM:** We fit LMM using the GEMMA algorithm [16].

2. **BVSR:** We fit BVSR by fixing $\rho = 1$ in BSLMM using our software. This gives slightly better results, and is faster than the BVSR software piMASS (version 0.90) [13], in all examples considered here.

3. **LMM-Bayes:** We fit this by fixing $\rho = 0$ in BSLMM using our software.

4. **Bayesian Lasso:** This [17] assumes a double-exponential prior for each coefficient β_i in (1):

$$\beta_i|\lambda \sim \text{DE}(0, \lambda^{-1}), \quad \lambda^2 \sim \text{Gamma}(\kappa_1, \kappa_2), \quad \tau^{-1} \sim \text{IG}(\kappa_3, \kappa_4), \tag{51}$$

   where DE denotes the double exponential (Laplace) distribution with mean 0 and scale parameter λ^{-1}, and Gamma denotes a Gamma distribution with shape and rate parameters. We set $\kappa_1 = 0.55, \kappa_2 = 10^{-6}, \kappa_3 = 1/2$ and $\kappa_4 = 1/2$ following previous studies [11, 18]. We use a conjugate Gamma prior for λ^2 as in [17] instead of a Beta prior for $\lambda/100$ as in [11]. We used the R package BLR [11] to sample from the posterior distribution of $\boldsymbol{\beta}$.

5. **BayesA-Flex:** This assumes a scaled t-distribution for each coefficient β_i in (1):

$$\beta_i|\sigma \sim t(0, \nu, \sigma^2), \quad \sigma^2 \sim \text{IG}(\kappa_1, \kappa_2), \quad \tau^{-1} \sim \text{IG}(\kappa_3, \kappa_4), \tag{52}$$

   where IG stands for the inverse gamma distribution. Following previous studies, we set the degree of freedom parameter ν to 4 [19–21] and set $\kappa_3 = 1/2$ and $\kappa_4 = 1/2$ [11]. We also consider the

posterior distribution where $\kappa_1 \to 0$ and $\kappa_2 \to 0$. The above model is similar to BayesA [19], but with a key difference in the way the scaling parameter σ^2 is treated: BayesA fixes σ^2 to some pre-specified value, whereas here we specify a prior for σ^2 and allow it to be estimated from the data (and hence the name "BayesA-Flex"). Using BayesA in this data set gives poor results [10] (and data not shown); but estimating the scaling parameter greatly improves prediction performance. We modified the R package BLR [11] to obtain posterior samples from this model. The modified code is freely available online.

6. **BayesCπ:** we fit this using the online software GenSel [21].

7. **BSLMM-EB:** we fit this using our BSLMM software, fixing σ_b^2 to the REML estimate $\hat{\sigma}_b^2$ from the null model (i.e. LMM). This approximation avoids updating σ_b^2 in each iteration of the MCMC, and is one of the several approximation strategies used by previous studies to alleviate the computation burden of models similar to BSLMM [9,22]. Intuitively, by fixing the variance component to its null estimate, BSLMM-EB discourages the inclusion of large effect SNPs into the sparse effects term and risks underestimating their effect sizes. Therefore, this approximation may reduce the prediction performance of BSLMM, especially when there are large effect SNPs. We confirm this in the real data set.

For all MCMC based methods except for BayesCπ, we run 2.1 million iterations with the first 0.1 million iterations as burn-in steps. For BayesCπ, due to web server restriction, we run 1.1 million iterations with the first 0.1 million iterations as burn-in.

References

1. Yang J, Benyamin B, McEvoy BP, Gordon S, Henders AK, et al. (2010) Common SNPs explain a large proportion of the heritability for human height. Nat Genet 42: 565-569.

2. Howie BN, Donnelly P, Marchini J (2009) A flexible and accurate genotype imputation method for the next generation of genome-wide association studies. PLOS Genet 5: e1000529.

3. Barber MJ, Mangravite LM, Hyde CL, Chasman DI, Smith JD, et al. (2010) Genome-wide association of lipid-lowering response to statins in combined study populations. PLOS One 5: e9763.

4. Simon JA, Lin F, Hulley SB, Blanche PJ, Waters D, et al. (2006) Phenotypic predictors of response to *Simvastatin* therapy among African-Americans and Caucasians: The cholesterol and pharmacogenetics (CAP) study. Am J Cardiol 97: 843-850.

5. Albert MA, Danielson E, Rifai N, Ridker PM (2001) Effect of statin therapy on C-reactive protein levels: the pravastatin inflammation/CRP evaluation (PRINCE): a randomized trial and cohort study. J Am Med Assoc 286: 64-70.

6. The Wellcome Trust Case Control Consortium (2007) Genome-wide association study of 14,000 cases of seven common diseases and 3,000 shared controls. Nature 447: 661-678.

7. Guan Y, Stephens M (2008) Practical issues in imputation-based association mapping. PLOS Genet 4: e1000279.

8. Valdar W, Solberg LC, Gauguier D, Burnett S, Klenerman P, et al. (2006) Genome-wide genetic association of complex traits in heterogeneous stock mice. Nat Genet 38: 879-887.

9. Lee SH, van der Werf JHJ, Hayes BJ, Goddard ME, Visscher PM (2008) Predicting unobserved phenotypes for complex traits from whole-genome SNP data. PLOS Genet 4: e1000231.

10. Legarra A, Robert-Granié C, Manfredi E, Elsen JM (2008) Performance of genomic selection in mice. Genetics 180: 611-618.

11. de los Campos G, Naya H, Gianola D, Crossa J, Legarra A, et al. (2009) Predicting quantitative traits with regression models for dense molecular markers and pedigree. Genetics 182: 375-385.

12. Valdar W, Solberg LC, Gauguier D, Cookson WO, Rawlins JNP, et al. (2006) Genetic and environmental effects on complex traits in mice. Genetics 174: 959-984.

13. Guan Y, Stephens M (2011) Bayesian variable selection regression for genome-wide association studies, and other large-scale problems. Ann Appl Stat 5: 1780-1815.

14. Wakefield J (2009) Bayes factors for genome-wide association studies: Comparison with p-values. Genet Epidemiol 33: 79-86.

15. Stephens M, Balding DJ (2009) Bayesian statistical methods for genetic association studies. Nat Rev Genet 10: 681-690.

16. Zhou X, Stephens M (2012) Genome-wide efficient mixed-model analysis for association studies. Nat Genet 44: 821-824.

17. Park T, Casella G (2008) The Bayesian lasso. J Am Stat Assoc 103: 681-686.

18. Makowsky R, Pajewski NM, Klimentidis YC, Vazquez AI, Duarte CW, et al. (2011) Beyond missing heritability: Prediction of complex traits. PLOS Genet 7: e1002051.

19. Meuwissen THE, Hayes BJ, Goddard ME (2001) Prediction of total genetic value using genome-wide dense marker maps. Genetics 157: 1819-1829.

20. Hayes BJ, Pryce J, Chamberlain AJ, Bowman PJ, Goddard ME (2010) Genetic architecture of complex traits and accuracy of genomic prediction: Coat colour, milk-fat percentage, and type in Holstein cattle as contrasting model traits. PLOS Genet 6: e1001139.

21. Habier D, Fernando RL, Kizilkaya K, Garrick DJ (2011) Extension of the Bayesian alphabet for genomic selection. BMC Bioinformatics 12: 186.

22. Segura V, Vilhjálmsson BJ, Platt A, Korte A, Ümit Seren, et al. (2012) An efficient multi-locus mixed-model approach for genome-wide association studies in structured populations. Nat Genet 44: 825-830.
