# Supplementary material for: Polygenic Modeling with Bayesian Sparse Linear Mixed Models
Source: PLoS Genet. 2013 Feb 7;9(2):e1003264. doi: 10.1371/journal.pgen.1003264 (PMC3567190; doi:10.1371/journal.pgen.1003264)
Supplement: Text S2 — Detailed MCMC Strategy for BSLMM. (PDF) [file pgen.1003264.s009.pdf]

Text S2 Detailed MCMC Strategy for BSLMM

To simplify notation, we assume in this section that $\mathbf{y}$ is centered. We use Markov chain Monte Carlo to obtain posterior samples of $(h, \rho, \pi, \boldsymbol{\gamma})$ on the product space $(0,1) \times (0,1) \times (0,1) \times \{0,1\}^p$, which is given by

$$P(h, \rho, \pi, \boldsymbol{\gamma}|\mathbf{y}) \propto P(\mathbf{y}|h, \rho, \pi, \boldsymbol{\gamma}) P(h) P(\rho) P(\boldsymbol{\gamma}|\pi) P(\pi). \tag{53}$$

In the above equation, we explored the fact that the parameters $\tilde{\boldsymbol{\beta}}$, $\mathbf{u}$ and τ can be integrated out analytically to compute the marginal likelihood $P(\mathbf{y}|h, \rho, \pi, \boldsymbol{\gamma})$. The marginal likelihood is

$$P(\mathbf{y}|h, \rho, \pi, \boldsymbol{\gamma}) \propto |\mathbf{H}|^{-\frac{1}{2}} |\sigma_a^{-2}\mathbf{\Omega}|^{\frac{1}{2}} (\mathbf{y}^T\mathbf{P}\mathbf{y})^{-\frac{n}{2}}, \tag{54}$$

where $\mathbf{H}(\sigma_b^2) = \sigma_b^2\mathbf{K} + \mathbf{I}_n$, $\mathbf{\Omega}(\sigma_a^2, \sigma_b^2, \gamma) = (\mathbf{X}_\gamma^T\mathbf{H}^{-1}\mathbf{X}_\gamma + \sigma_a^{-2}\mathbf{I}_{|\gamma|})^{-1}$, $\mathbf{P}(\sigma_a^2, \sigma_b^2, \gamma) = \mathbf{H}^{-1} - \mathbf{H}^{-1}\mathbf{X}_\gamma\mathbf{\Omega}\mathbf{X}_\gamma^T\mathbf{H}^{-1}$. Notice again that σ_a^2 is a function of h, ρ and π, while σ_b^2 is a function of h and ρ.

To efficiently evaluate the marginal likelihood, we perform an eigen decomposition of the relatedness matrix $\mathbf{K} = \mathbf{U}\mathbf{D}\mathbf{U}^T$ at the beginning of the MCMC, where $\mathbf{U}$ is the matrix of eigen vectors and $\mathbf{D}$ is a diagonal matrix of eigen values. We transform both the phenotype vector and the genotype matrix by multiplying the eigen matrix and calculate $\mathbf{U}^T\mathbf{y}$ and $\mathbf{U}^T\mathbf{X}$. Afterwards, as has been shown previously, the calculations of the determinant and the inverse of matrix $\mathbf{H}$, as well as the vector-matrix-vector form $\mathbf{y}^T\mathbf{P}\mathbf{y}$, in each iteration of the MCMC, are easy to perform [1,2].

We use a standard Metropolis-Hastings algorithm to draw posterior samples of the hyper-parameters $(h, \rho, \pi, \boldsymbol{\gamma})$ based on the above marginal likelihood. Following [3], we use a rank based proposal distribution for $\boldsymbol{\gamma}$, and use random walk proposals based on uniform distributions for h, ρ and $\log(\pi)$. In particular, we first obtain single-SNP p values using a standard LMM with GEMMA algorithm [2], and then rank SNPs based on these p values from small to large. Our aim is to use a proposal distribution for $\boldsymbol{\gamma}$ that puts more weights on SNPs that are ranked higher by the single SNP tests, and to do this we consider a mixture distribution $Q_p = 0.3U_p + 0.7G_p$, where U_p is a uniform distribution on $1, \cdots, p$ and G_p is a geometric distribution truncated to $1, \cdots, p$ with its parameter chosen to give a mean of 2000. We denote $\boldsymbol{\gamma}^+ = \{i : \gamma_i = 1\}$ and we propose the new $\boldsymbol{\gamma}$ by randomly choose one of the following steps:

- add a covariate with probability 0.4: generate r from Q_p until the covariate with rank r is not in $\boldsymbol{\gamma}^+$, then add this covariate to $\boldsymbol{\gamma}^+$
- remove a covariate with probability 0.4: pick a covariate in $\boldsymbol{\gamma}^+$ uniformly at random and remove it from $\boldsymbol{\gamma}^+$
- switch a pair of covariates with probability 0.2: pick up two covariates by the above two steps, and switch their indicator values

For the other hyper-parameters, we update $\log(\pi)$ by adding a random variable from U(-0.05, 0.05) to the current value, and update h and ρ by adding a random variable from U(-0.1, 0.1) to the current values. New values of h and ρ that lie outside the boundary [0,1] are reflected back.

In addition to the above local proposal distributions, we also use a "small world proposal" which improves theoretical MCMC convergence [4]. In brief, with probability 0.33 in each iteration, we make a longer-range proposal by compounding many local moves, where the number of compounded local moves is draw uniformly from 1 to 20.

For each sampled values of $(h, \rho, \pi, \boldsymbol{\gamma})$, we further obtain samples of τ and $\tilde{\boldsymbol{\beta}}$ using the conditional distributions $P(\tau|\mathbf{y}, h, \rho, \pi, \boldsymbol{\gamma})$ and $P(\tilde{\boldsymbol{\beta}}|\mathbf{y}, h, \rho, \pi, \boldsymbol{\gamma}, \tau)$ listed below:

$$\tau|\mathbf{y}, h, \rho, \pi, \boldsymbol{\gamma} \sim \text{Gamma}(\frac{n}{2}, \frac{\mathbf{y}^T\mathbf{P}\mathbf{y}}{2}), \tag{55}$$

$$\tilde{\boldsymbol{\beta}}_{\boldsymbol{\gamma}}|\mathbf{y}, h, \rho, \pi, \boldsymbol{\gamma}, \tau \sim \text{MVN}_{|\gamma|}(\mathbf{\Omega}\mathbf{X}_\gamma^T\mathbf{H}^{-1}\mathbf{y}, \tau^{-1}\mathbf{\Omega}), \tag{56}$$

$$\tilde{\boldsymbol{\beta}}_{-\boldsymbol{\gamma}}|\mathbf{y}, h, \rho, \pi, \boldsymbol{\gamma}, \tau \sim \boldsymbol{\delta}_0. \tag{57}$$

Afterwards, we sample $\mathbf{u}$ based the conditional distribution $P(\mathbf{u}|\mathbf{y}, h, \rho, \pi, \boldsymbol{\gamma}, \tau, \tilde{\boldsymbol{\beta}})$:

$$\mathbf{u}|\mathbf{y}, h, \rho, \pi, \boldsymbol{\gamma}, \tau, \tilde{\boldsymbol{\beta}} \sim \mathrm{MVN}_n(\sigma_b^2 \mathbf{K}\mathbf{H}^{-1}(\mathbf{y} - \mathbf{X}_\gamma \tilde{\boldsymbol{\beta}}_\gamma), \sigma_b^2 \mathbf{K}\mathbf{H}^{-1}\tau^{-1}). \tag{58}$$

However, we do not sample $\mathbf{u}$ directly from the above n-dimensional multivariate normal distribution. Instead, we sample $\mathbf{U}^T\mathbf{u}$ (and we never need to obtain $\mathbf{u}$), as the conditional distribution of each element in $\mathbf{U}^T\mathbf{u}$ is a normal:

$$\mathbf{U}^T\mathbf{u}|\mathbf{y}, h, \rho, \pi, \boldsymbol{\gamma}, \tau, \tilde{\boldsymbol{\beta}} \sim \mathrm{MVN}_n(\sigma_b^2 \mathbf{D}(\sigma_b^2\mathbf{D} + \mathbf{I})^{-1}(\mathbf{U}^T\mathbf{y} - \mathbf{U}^T\mathbf{X}_\gamma \tilde{\boldsymbol{\beta}}_\gamma), \sigma_b^2 \mathbf{D}(\sigma_b^2\mathbf{D} + \mathbf{I})^{-1}\tau^{-1}). \tag{59}$$

where the covariance matrix is diagonal.

For each sampled value of ($\tilde{\boldsymbol{\beta}}$, $\mathbf{u}$, τ), we obtain samples of PVE and PGE based on equations (13) and (14).

When required (e.g. for evaluating RPG in simulation studies), in the special case $\mathbf{K} = \mathbf{X}\mathbf{X}^T/p$, we also obtain the (approximate) posterior mean of $\boldsymbol{\alpha}$ in the alternative model formulation (46)-(49). This is achieved without sampling $\boldsymbol{\alpha}$ in each iteration using the fact that the full conditional distribution of $\boldsymbol{\alpha}$ given other sampled values is

$$\boldsymbol{\alpha}|\mathbf{y}, h, \rho, \pi, \boldsymbol{\gamma}, \tau, \tilde{\boldsymbol{\beta}} \sim \mathrm{MVN}_n(\sigma_b^2 p^{-1}\mathbf{X}^T\mathbf{H}^{-1}(\mathbf{y} - \mathbf{X}_{\boldsymbol{\gamma}}\tilde{\boldsymbol{\beta}}_{\boldsymbol{\gamma}}), \sigma_b^2(p^{-1}\mathbf{I}_p - p^{-2}\sigma_b^2\mathbf{X}^T\mathbf{H}^{-1}\mathbf{X})\tau^{-1}), \tag{60}$$

which leads to the Rao-Blackwellised approximation for the posterior mean of $\boldsymbol{\alpha}$:

$$\hat{\boldsymbol{\alpha}} = \frac{1}{T}\sum_{t=1}^{T} \mathrm{E}(\boldsymbol{\alpha}|\mathbf{y}, h^{(t)}, \rho^{(t)}, \pi^{(t)}, \boldsymbol{\gamma}^{(t)}, \tau^{(t)}, \tilde{\boldsymbol{\beta}}^{(t)}) = \frac{1}{p}\mathbf{X}^T \frac{1}{T}\sum_{t=1}^{T}(\sigma_b^{(t)})^2(\mathbf{H}^{(t)})^{-1}(\mathbf{y} - \mathbf{X}_{\boldsymbol{\gamma}^{(t)}}\tilde{\boldsymbol{\beta}}^{(t)}_{\boldsymbol{\gamma}^{(t)}}), \tag{61}$$

where T is the total number of MCMC iterations, and the superscript (t) denotes the tth MCMC sample. Notice that we only need to do the p dimensional matrix-vector multiplication once at the end.

When $|\gamma|$ is large, the most time consuming part of our MCMC scheme for fitting BSLMM and BVSR is the calculation of Ω. The per-iteration computation time of the above algorithm is comparable to that of BVSR [3] with linear complexity in the number of individuals but quadratic complexity in $|\boldsymbol{\gamma}|$. In practice, to reduce the computation burden, we set a maximal value for $|\boldsymbol{\gamma}|$ (300 for simulations and the two human data sets, 600 for the mouse data set). Setting the maximal value to a larger number (600) in simulations improves results only subtly, even for scenarios where a large number of causal SNPs is present.

References

1. Lippert C, Listgarten J, Liu Y, Kadie CM, Davidson RI, et al. (2011) FaST linear mixed models for genome-wide association studies. Nat Methods 8: 833-835.

2. Zhou X, Stephens M (2012) Genome-wide efficient mixed-model analysis for association studies. Nat Genet 44: 821-824.

3. Guan Y, Stephens M (2011) Bayesian variable selection regression for genome-wide association studies, and other large-scale problems. Ann Appl Stat 5: 1780-1815.

4. Guan Y, Krone SM (2007) Small-world MCMC and convergence to multi-modal distributions: From slow mixing to fast mixing. Ann Appl Probab 17: 284–304.
