# Supplementary material for: Polygenic Modeling with Bayesian Sparse Linear Mixed Models
Source: PLoS Genet. 2013 Feb 7;9(2):e1003264. doi: 10.1371/journal.pgen.1003264 (PMC3567190; doi:10.1371/journal.pgen.1003264)
Supplement: Text S3 — The Probit BSLMM and Binary Traits. (PDF) [file pgen.1003264.s010.pdf]

Text S3 The Probit BSLMM and Binary Traits

MCMC strategy

We use "probit BSLMM" to refer to a BSLMM with a probit link to model binary traits:

$$P(y_i = 1|\mathbf{x}_i, \tilde{\boldsymbol{\beta}}, u_i) = 1 - P(y_i = 0|\mathbf{x}_i, \tilde{\boldsymbol{\beta}}, u_i) = \Phi(\mu + \mathbf{x}_i^T \tilde{\boldsymbol{\beta}} + u_i) \quad (i = 1, \cdots, n), \tag{62}$$

where y_i is the binary trait for ith individual, $\mathbf{x}_i$ is the p-vector of genotypes for ith individual, u_i is ith element of random effects vector $\mathbf{u}$ and Φ is the cumulative distribution function (CDF) of the standard normal distribution. Following [1], we introduce a vector of auxiliary variables $\mathbf{z}$ and obtain the equivalent latent variable model as:

$$y_i = \begin{cases} 1 & \text{if } z_i > 0 \\ 0 & \text{if } z_i \leq 0 \end{cases}, \tag{63}$$

$$z_i = \mu + \mathbf{x}_i^T \tilde{\boldsymbol{\beta}} + u_i + \epsilon_i \quad \epsilon_i \sim \mathrm{N}(0, 1), \tag{64}$$

where z_i is ith element of vector $\mathbf{z}$.

We use the same prior specifications for the hyper-parameters as described in the main text (except that $\tau = 1$ here). We use a similar MCMC strategy as described in Text S2 to sample posteriors, with an additional step to sample the posteriors of the latent variables $\mathbf{z}$ using the conditional distribution $P(\mathbf{z}|\mathbf{y}, \boldsymbol{\gamma}, \tilde{\boldsymbol{\beta}}, \mathbf{u})$:

$$z_i|y_i = 1, \tilde{\boldsymbol{\beta}}, u_i \sim \mathrm{N}(\mu + \mathbf{x}_{\gamma i}^T \tilde{\boldsymbol{\beta}} + u_i, 1) \quad \text{left truncated at 0}, \tag{65}$$

$$z_i|y_i = 0, \tilde{\boldsymbol{\beta}}, u_i \sim \mathrm{N}(\mu + \mathbf{x}_{\gamma i}^T \tilde{\boldsymbol{\beta}} + u_i, 1) \quad \text{right truncated at 0}. \tag{66}$$

We denote $\bar{z}$ as the sample mean of $\mathbf{z}$, or $\bar{z} = \frac{1}{n} \sum_{i=1}^n z_i$. Conditional on the latent variables $\mathbf{z}$, the posterior sampling for the hyper-parameters $(h, \rho, \pi, \boldsymbol{\gamma})$ is based on the marginal likelihood $P(h, \rho, \pi, \boldsymbol{\gamma}|\mathbf{z})$, which is slightly different from that in Text S2 as we do not integrate out τ here:

$$P(\mathbf{z}|h, \rho, \pi, \boldsymbol{\gamma}) \propto |\mathbf{H}|^{-\frac{1}{2}} |\sigma_a^{-2} \boldsymbol{\Omega}|^{\frac{1}{2}} e^{-\frac{1}{2}(\mathbf{z} - \mathbf{1}_n \bar{z})^T \mathbf{P} (\mathbf{z} - \mathbf{1}_n \bar{z})}. \tag{67}$$

After obtaining the posterior samples of the hyper-parameters, we sample the posteriors of $\tilde{\boldsymbol{\beta}}$ and $\mathbf{u}$ using conditional distributions identical to those listed in Text S2 by setting $\tau = 1$. Finally, we sample μ based on the conditional distribution $P(\mu|\mathbf{z}, \boldsymbol{\gamma}, \tilde{\boldsymbol{\beta}}, \mathbf{u})$:

$$\mu|\mathbf{z}, \boldsymbol{\gamma}, \tilde{\boldsymbol{\beta}}, \mathbf{u} \sim \mathrm{N}(\frac{1}{n} \mathbf{1}_n^T (\mathbf{z} - \mathbf{X}_{\boldsymbol{\gamma}} \tilde{\boldsymbol{\beta}}_{\boldsymbol{\gamma}} - \mathbf{u}), \frac{1}{n}). \tag{68}$$

For efficient calculation of the above marginal likelihood function $P(\mathbf{z}|h, \rho, \pi, \boldsymbol{\gamma})$, we use the same strategy as described in Text S2. However, as a transformation of the latent vector $\mathbf{z}$ to $\mathbf{U}^T \mathbf{z}$ as well as transformations of $\mathbf{U}^T \mathbf{u}$ and $\mathbf{U}^T \mathbf{X} \tilde{\boldsymbol{\beta}}$ back to $\mathbf{u}$ and $\mathbf{X} \tilde{\boldsymbol{\beta}}$ are needed in every Gibbs iteration, the per-iteration computational cost of the probit BSLMM increases quadratically with the number of individuals.

Application to Mouse Data

To generate a binary data set on which to illustrate the probit BSLMM and compare its performance with BSLMM, we use the mouse data from the main text, transforming the quantitative values of the three traits into binary values by assigning the half individuals with higher quantitative values to 1 and the other half to 0. We consider two different approaches here: (linear) BSLMM and probit BSLMM. The BSLMM can be viewed as a first order approximation to its probit counterpart. We use Brier score in the test sample to evaluate prediction performance. For BSLMM, we threshold the predicted probability

values that are above 1 to be exact 1 and those below 0 to be exact 0. We contrast the performance of the probit BSLMM against BSLMM by calculating the Brier score difference, where a positive value indicates worse performance than BSLMM.

Figure S6 shows Brier score differences for the three traits. Interestingly, for the three traits here, treating binary values as quantitative traits using BSLMM works better than modeling them directly using the probit BSLMM. This may partly reflect numerical inaccuracies due to the greater computational burden of fitting the probit BSLMM.

Correction factor for estimating PVE for case-control data

Here, we provide an alternative way to derive the correction factor, that appeared in [2], for transforming PVE estimate in the observed scale back to that in the liability scale. Our approach is based on Taylor series approximation. To simplify notation, we denote $k_p = P_p(y_i = 1)$ as the case proportion in the population, $k_a = P_a(y_i = 1)$ as the case proportion in the ascertained case-control sample, Φ as the normal CDF (cumulative distribution function), ϕ as the normal PDF (probability distribution function), μ_p satisfies $\Phi(\mu_p) = k_p$, μ_a satisfies $\Phi(\mu_a) = k_a$, and $z_p = \phi(\mu_p)$.

First, we assume, following [2], a probit model on the population scale:

$$P_p(y_i = 1|\mathbf{x}_i, \tilde{\boldsymbol{\beta}}, u_i) = 1 - P_p(y_i = 0|\mathbf{x}_i, \tilde{\boldsymbol{\beta}}, u_i) = \Phi(\mu_p + \mathbf{x}_i^T\tilde{\boldsymbol{\beta}} + u_i) \quad (i = 1, \cdots, N), \tag{69}$$

where N is the population sample size.

The conditional distribution in the ascertained case-control sample can be derived by Bayes theorem

$$P_a(y_i = 1|\mathbf{x}_i, \tilde{\boldsymbol{\beta}}, u_i) = \frac{P_a(\mathbf{x}_i, u_i|y_i = 1, \tilde{\boldsymbol{\beta}})P_a(y_i = 1|\tilde{\boldsymbol{\beta}})}{P_a(\mathbf{x}_i, u_i|\tilde{\boldsymbol{\beta}})} \quad (i = 1, \cdots, n), \tag{70}$$

where n is the case-control sample size.

We notice that $P_a(\mathbf{x}_i, u_i|y_i = 1, \tilde{\boldsymbol{\beta}}) = P_p(\mathbf{x}_i, u_i|y_i = 1, \tilde{\boldsymbol{\beta}})$ holds for ideal case-control studies, as cases in the ascertained sample are selected randomly from all cases in the population. We assume further $P_p(y_i = 1|\tilde{\boldsymbol{\beta}}) \approx P_p(y_i = 1) = k_p$ and $P_a(y_i = 1|\tilde{\boldsymbol{\beta}}) \approx P_a(y_i = 1) = k_a$, that the probability of being a case does not depend on parameters, an assumption commonly made (see e.g. [3]) and likely hold when parameters are close to their true values. We further denote a normalizing constant $Z = \frac{P_a(\mathbf{x}_i, u_i|\tilde{\boldsymbol{\beta}})}{P_p(\mathbf{x}_i, u_i|\tilde{\boldsymbol{\beta}})}$, and we have

$$P_a(y_i = 1|\mathbf{x}_i, \tilde{\boldsymbol{\beta}}, u_i) \approx \frac{1}{Z}\frac{k_a}{k_p}\Phi(\mu_p + \mathbf{x}_i^T\tilde{\boldsymbol{\beta}} + u_i), \tag{71}$$

and similarly

$$P_a(y_i = 0|\mathbf{x}_i, \tilde{\boldsymbol{\beta}}, u_i) \approx \frac{1}{Z}\frac{1 - k_a}{1 - k_p}(1 - \Phi(\mu_p + \mathbf{x}_i^T\tilde{\boldsymbol{\beta}} + u_i)), \tag{72}$$

which give the normalizing constant

$$Z = \frac{k_a}{k_p}\Phi(\mu_p + \mathbf{x}_i^T\tilde{\boldsymbol{\beta}} + u_i) + \frac{1 - k_a}{1 - k_p}(1 - \Phi(\mu_p + \mathbf{x}_i^T\tilde{\boldsymbol{\beta}} + u_i)). \tag{73}$$

We expand the above two likelihoods using Taylor series expansion with respect to $\mathbf{x}_i^T\tilde{\boldsymbol{\beta}} + u_i$ at 0. If we use the linear term only for approximation, we obtain

$$P_a(y_i = 1|\mathbf{x}_i, \tilde{\boldsymbol{\beta}}, u_i) \approx k_a + \frac{k_a(1 - k_a)z_p}{k_p(1 - k_p)}(\mathbf{x}_i^T\tilde{\boldsymbol{\beta}} + u_i), \tag{74}$$

$$P_a(y_i = 0|\mathbf{x}_i, \tilde{\boldsymbol{\beta}}, u_i) \approx 1 - k_a - \frac{k_a(1 - k_a)z_p}{k_p(1 - k_p)}(\mathbf{x}_i^T\tilde{\boldsymbol{\beta}} + u_i). \tag{75}$$

In other words, the expected value of individual binary label can be approximated by

$$E(y_i) = P_a(y_i = 1|\mathbf{x}_i, \tilde{\boldsymbol{\beta}}, u_i) \approx k_a + \frac{k_a(1-k_a)z_p}{k_p(1-k_p)}(\mathbf{x}_i^T\tilde{\boldsymbol{\beta}} + u_i), \tag{76}$$

which suggests using a linear mixed model to treat binary values as quantitative traits to infer the parameters. The estimated PVE on the observed scaling using a linear mixed model is

$$\hat{\text{PVE}}_o = (\frac{k_a(1-k_a)}{k_p(1-k_p)}z_p)^2\frac{V(\mathbf{X}\hat{\tilde{\boldsymbol{\beta}}} + \hat{\mathbf{u}})}{V(\mathbf{y})} = \frac{k_a(1-k_a)z_p^2}{k_p^2(1-k_p)^2}V(\mathbf{X}\hat{\tilde{\boldsymbol{\beta}}} + \hat{\mathbf{u}}) \approx \frac{k_a(1-k_a)z_p^2}{k_p^2(1-k_p)^2}\text{PVE}_l, \tag{77}$$

where $\hat{\text{PVE}}_o$ is the PVE estimate on the observed scale, and PVE_l is the true PVE on the liability scale.

Therefore, we can use the correction factor $\frac{k_p^2(1-k_p)^2}{k_a(1-k_a)z_p^2}$ to transform the PVE estimate on the observed scale back to that on the liability scale.

References

1. Albert JH, Chib S (1993) Bayesian anal of binary and polychotomous response data. J Am Stat Assoc 88: 669-679.

2. Lee SH, Wray NR, Goddard ME, Visscher PM (2011) Estimating missing heritability for disease from genome-wide association studies. Am J Hum Genet 88: 294-305.

3. Imbens GW (1992) An efficient method of moments estimator for discrete choice models with choice-based sampling. Econometrica 60: 1187-1214.
